# Extracellular condensates (ECs) are endogenous modulators of HIV transcription and latency reactivation

**DOI:** 10.1038/s41380-025-03354-w

**Published:** 2025-11-29

**Authors:** Wasifa Naushad, Lakmini S. Premadasa, Vyshnavi Tallapaneni, Bryson C. Okeoma, Ashok Chaudhary, Jack T. Stapleton, Mahesh Mohan, Chioma M. Okeoma

**Affiliations:** 1 Department of Pathology, Microbiology & Immunology, New York Medical College, Valhalla, NY USA; 2 Host Pathogen Interaction Program, Southwest National Primate Research Center, Texas Biomedical Research Institute, San Antonio, TX 78227-5302 USA; 3 Department of Internal Medicine, Carver College of Medicine, University of Iowa, 200 Hawkins Drive, Iowa City, IA 52242-1109 USA; 4 Medical Service, Iowa City Veterans Affairs Medical Center, University of Iowa, 604 Highway 6, Iowa City, IA 52246-2208 USA; 5 Lovelace Biomedical Institute, Albuquerque, NM 87108-5127 USA

**Keywords:** Molecular biology, Neuroscience

## Abstract

The persistence of HIV latent reservoir is the major challenge to HIV cure because latent viruses serve as sources for viral rebound upon ART cessation. Mechanisms regulating viral persistence are not well understood; thus, there is a compelling need for research focusing on addressing the knowledge gap related to HIV persistence. The present study focuses on the effect of extracellular condensates (ECs) on latent HIV/SIV reactivation in the brain in the context of HIV infection using the SIV-infected rhesus macaque model. We used in vitro model systems of post-integration latency and primary peripheral blood mononuclear cells isolated from HIV-infected ART-suppressed donors to explore the role of basal ganglia (BG) isolated extracellular condensates (ECs) in reprogramming HIV latent cells. We found that BG ECs from uninfected macaques (VEH) and SIV infected macaques (VEH | SIV) activated latent HIV transcription in various model systems. VEH | SIV ECs significantly increased the expression and production of viral antigen in latently infected cells. Activation of viral transcription, antigen expression, and latency reactivation was inhibited by ECs from the brain of macaques treated with Delta-9-tetrahydrocannabinol (THC) and infected with SIV (THC | SIV). Virus produced by latently infected cells treated with VEH | SIV ECs potentiated cell-cell and cell-free HIV transmission. VEH | SIV ECs also reversed dexamethasone-mediated inhibition of HIV transcription while TNFα-mediated reactivation of latency was reversed by THC | SIV ECs. Transcriptome and secretome analyses of total RNA and supernatants from latently infected cells treated with ECs revealed significant alterations in gene expression and cytokine secretion. THC | SIV ECs increased secretion of Th2 and decreased secretion of proinflammatory cytokines. Most strikingly, while VEH/SIV ECs robustly induced expression of HIV RNA in latently HIV-infected cells, increased the frequency of HIV gag p24 expressing cells in HIV-infected CD4 + T cells within PBMCs, and production of extracellular HIV gag p24, long-term low-dose THC administration enriched ECs with anti-inflammatory cargo that significantly diminished their ability to reactivate latent HIV, an indication that ECs are endogenous host factors that may regulate HIV persistence.

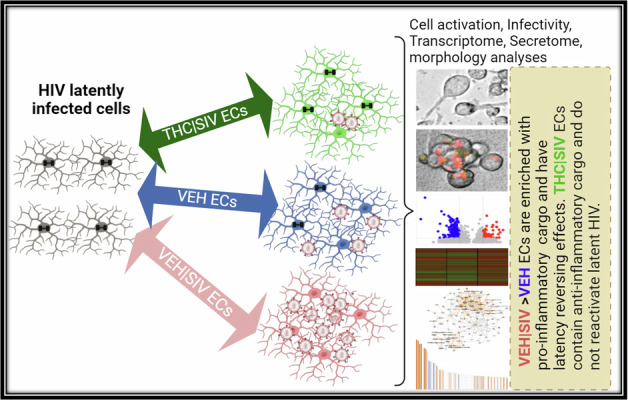

## Highlights

- ECs isolated from SIV infected rhesus macaques (VEH | SIV ECs) are positive regulators of LTR-dependent HIV transcription and production of infectious viral particles in vitro.
- ECs isolated from THC treated SIV infected rhesus macaques (THC | SIV ECs) prevents the transcription and reactivation of HIV in latently infected cells and prevents production of HIV gag p24 and viral particles in vitro.
- ECs reprogram host transcriptome and secretome in manners that suppress or promote reactivation of latent HIV reservoir.
- These observations were made in HIV latently infected cell lines, including T cell lines, monocytes, microglia, as well as in primary peripheral blood mononuclear cells (PBMC) isolated from HIV-infected ART-suppressed donors.

## Introduction

Finding a cure for HIV requires rigorous study of tissue reservoirs and the extracellular host factors that regulate HIV persistence despite better access to and effectiveness of antiretroviral therapy (ART). The central nervous system (CNS) is a unique site where HIV persists. Circulating CD4 T cells are the major HIV reservoir in the periphery, and these cells migrate into the CNS and may contribute to HIV persistence in the brain [1]. Additionally, myeloid cells (monocytes, macrophages, and microglia) are also HIV targets/reservoirs. HIV may persist in brain myeloid cells [2–6] and these cells may contribute to the size of the latent reservoir, as revealed by the presence of HIV DNA in postmortem brains from people with HIV (PWH) [7, 8]. However, the host factors that regulate viral persistence in the CNS are not well understood. Thus, identifying the endogenous parameters that contribute to HIV persistence is of significant interest. We predict that extracellular condensates (ECs) may function as endogenous regulators of HIV persistence.

ECs are non-vesicular extracellular particles (NVEPs) non-lipid carriers of protein and nucleic acid cargo [9, 10] that may act in a paracrine manner to mediate cell to cell interaction, modulate host responses, and remodel cellular biological processes. Of particular interest and a question yet to be addressed is the link between endogenously secreted factors, such as ECs and their ability to reactivate latent HIV, especially given that they can be modified by the microenvironment, can be generated in vivo, and can trigger complex cellular responses. Indeed, it has been suggested that extracellular vesicles (EVs) in the brain may play a role in HIV pathogenesis [11].

Cannabis use is high among PWH and was associated with reduced immune activation [12], lower plasma HIV RNA [13] and most strikingly, significantly reduced proviral HIV DNA burden in multiple tissues [14]. Recently, our group showed that long-term low dose delta-9-tetrahydrocannabinol (THC) administration reduced neuroinflammation [15] and stimulated the release of blood EVs that induced divergent structural adaptations and signaling cues in chronically SIV-infected rhesus macaques [16]. We further showed that the basal ganglia (BG) contain ECs and EVs and that BG EVs serve as a vehicle with the potential to disseminate HIV/SIV and THC induced changes within the CNS [11]. Since the BG [17, 18] is a major HIV target/reservoir, these findings collectively underscore the need for further studies to investigate the effects of BG ECs and EVs on HIV persistence in the context of THC use.

The current study combined the SIVmac251-infected rhesus macaque model of HIV infection and pathogenesis, BG ECs, primary PBMCs isolated from HIV infected ART-suppressed donors, and in vitro model systems of post-integration latency to investigate the effect of ECs on the reactivation of latent HIV. We found specific effects of ECs in reactivating latent HIV in J-Lat -GFP, J-Lat Tat-GFP, U1 monocytic, and huglia (HC69) microglia cells, as well as HIV infected PBMCs. Moreover, we demonstrated EC-mediated alteration in the transcriptome and secretome of latently HIV-infected cells that were distinct in cells treated with VEH | SIV versus THC | SIV ECs. The transcriptome and secretome changes culminated in the alterations of important pathways, especially the NF-κB, replication factor C4 (RFC4), and CDK complexes, that are important regulators of HIV transcription, and reactivation of latent HIV. Altogether, our results demonstrate ECs as endogenous regulators of HIV latency with potential to control the size of viral reservoirs and persistence.

## Results

### Assessment of plasma and BG viral loads

Brain viral loads in the two groups ranged from 0.01 to 2.0 10^6^/mg (p = 0.100) RNA (Table 1) and were published previously [11]. ECs were isolated from BG tissues of VEH (n = 3), VEH | SIV (n = 4), THC | SIV (n = 4) RMs (Table 1). VEH represents uninfected control (n = 3). All THC/SIV RMs initiated twice daily THC injections (i/m) at 0.18 mg/kg two weeks before SIV infection and continued thereafter at 0.32 mg/kg until necropsy. All SIV-infected rhesus macaques were not treated with ART.

**Table 1:** Animal IDs, SIV inoculum, duration of infection, viral loads and brain histopathology in vehicle or delta-9-tetrahydrocannabinol (Δ9-THC) treated chronic SIV-infected and uninfected rhesus macaques.

Animal ID	SIV Inoculum	Duration of Infection	Plasma viral loads 10^6^/mL	Brain viral loads 10^6^/mg RNA	Brain Histopathology	Opportunistic Infections
**Chronic SIV-Infected and Vehicle treated Animals used for Isolation of ECs (Group 1)**						
IV95	SIVmac251	180	0.02	2.0	ND	ND
JD66	SIVmac251	180	0.04	0.2	ND	ND
JR36	SIVmac251	180	0.5	0.2	ND	ND
JH47	SIVmac251	180	2	0.07	ND	ND
**Chronic SIV-Infected and Δ**^**9**^**-THC treated Animals used for Isolation of ECs (Group 2)**						
JI45	SIVmac251	180	3	0.01	ND	ND
JT80	SIVmac251	180	1	0.04	ND	ND
JC85	SIVmac251	180	0.02	0.09	ND	ND
IV90	SIVmac251	180	0.02	0.06	ND	ND
**Uninfected Control Animals used for Isolation of ECs (Group 3)**						
IR97	NA	NA	NA	NA	NA	NA
IT18	NA	NA	NA	NA	NA	NA
GT18	NA	NA	NA	NA	NA	NA

*NA* not applicable, *ND* none detected.

### VEH | SIV BG ECs reactivate HIV latency in J Lat T cells

**Fig. 1:**
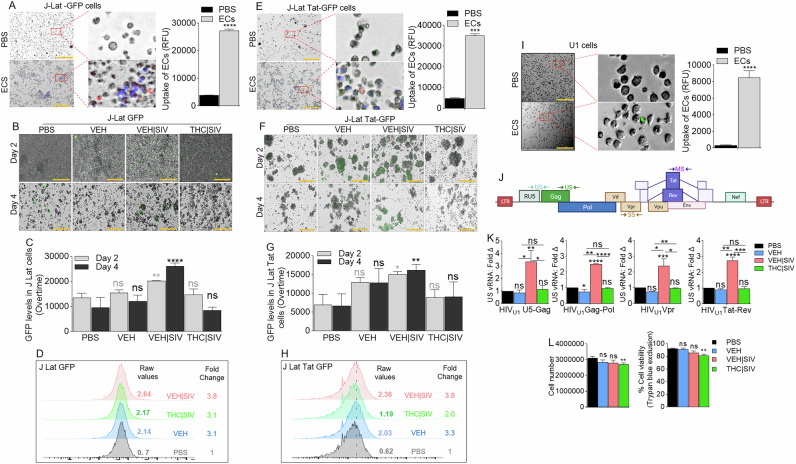
Basal ganglia derived ECs activate HIV latently infected cells. **A**) Representative microscopic images of DIR-labeled ECs internalized by J-Lat -GFP cells (left) and the level of internalized ECs (bars, right). **B**) Representative microscopic images of EC-treated J-Lat -GFP cells showing HIV reactivation (GFP expression, green) after 4 days of treatment. Representative data from day 4 treatment presented. **C**) The level of HIV reactivation analyzed by microscopy was quantified on days 2 and 4. **D**) Flow cytometry analysis of HIV reactivation (GFP expression, green) after 4 days of treatment. Numbers indicate raw values (left) or fold change (treatment/PBS) (right). **E**) Microscopic image of internalized DIR-labeled ECs by J-Lat Tat-GFP cells (left) and the level of internalized ECs (bars, right). **F**) Microscopic images of EC-treated J-Lat -GFP cells showing HIV reactivation (GFP expression, green) after 4 days of treatment. Representative data from day 4 treatment presented. **G**) The level of HIV reactivation analyzed by microscopy was quantified on days 2 and 4. **H**) Flow cytometry analysis of HIV reactivation (GFP expression, green) after 4 days of treatment. Numbers indicate raw values (left) or fold change (treatment/PBS) (right). **I**) Microscopic image of internalized SYTO RNASelect green fluorescent-labeled U1 cells (left) and the level of internalized ECs (bars, right). **J**) HIV genome showing the different HIV genes. Arrows indicate the positions of forward and reverse primers used in the analysis of HIV transcription. **K**) RT-qPCR analysis of the levels of intracellular viral RNA. Four HIV (unspliced [U5-Gag, Gag-Pol], singly spliced [Vpr], and multiply spliced [Tat-Rev]) transcripts were measured. **L**) Analysis of cell density following 4-day treatment with ECs as assessed by cell number (left) and trypan blue exclusion assay (right). All experiments were repeated three times. Statistical differences were assessed by ordinary one-way ANOVA with Tukey’s correction and by Binary Student’s t tests (Welch’s correction). **** p < 0.001, *** p < 0.005, ** p < 0.01, * p < 0.05, and ns = non-significant. Scale bars (horizontal orange lines) = 200 µm.

J-Lat GFP cells (Supplemental Fig. 1A, B) were treated with DiR-labeled PBS, or VEH, VEH | SIV, and THC | SIV ECs (Table 1). Internalization of ECs (Fig. 1A) and GFP expression as evidence of HIV reactivation (Fig. 1B, C) were analyzed at 24 h. Microscopic data showed that VEH | SIV ECs significantly increased GFP expression in J-Lat GFP cells, which was confirmed by flow cytometry with 3.8-fold change in GFP expression in VEH | SIV-treated cells compared to PBS treated cells (Fig. 1D). The effect of ECs on latent HIV reactivation was validated with J-Lat-Tat-GFP (Fig. 1E-H).

### VEH | SIV but not THC | SIV BG ECs reverse HIV-1 latency in U1 cells

HIV-1 latency reversal potential of VEH | SIV BG ECs was assessed using U1 monocytes as an in vitro model of post-integration latency [19, 20] (Supplemental Fig. 1A, C). ECs were internalized by U1 cells 24 h after treatment (Fig. 1I). The degree to which HIV expression is activated by ECs at different stages of HIV transcription was assessed using a panel of primers that amplify unspliced [U5-Gag, Gag-Pol], singly spliced [Vpr], and multiply spliced [Tat-Rev] transcripts (Table 2, Fig. 1J). Compared to PBS, VEH, and THC | SIV ECs, VEH | SIV ECs reversed HIV-1 latency as shown by the significant increases in all intracellular HIV RNA types (Fig. 1K). Since cell death in reactivated cells is a key mechanism to reduce HIV reservoir cells in vivo [21, 22], we showed that 24 h after treatment, THC | SIV but not VEH and VEH | SIV ECs modestly but significantly reduced U1 cell numbers (Fig. 1L**left**) and viability (Fig. 1L**right**).

**Table 2:** Primer sequences used in this study.

Gene	Forward sequence (5’→3’)	Reverse sequence (5’→3’)
GAPDH	CCCCTTCATTGACCTCAACTACA	CGCTCCTGGAGGATGGTGAT
HIV U5-Gag	TGTGTGCCCGTCTGTTGTGTGA	GAGTCCTGCGTCGAGAGAGCT
HIV Gag-Pol	TTCTTCAGAGCAGACCAGAGC	GCTGCCAAAGAGTGATCTGA
HIV Vpr	ACTTACGGGGATACTTGGGCAG	CTCCATTTCTTGCTCTCCTCTGTC
HIV Tat-Rev	GTGTGCCCGTCTGTTGTGTGACTCTGGTAAC	GCCTATTCTGCTATGTGACACCC
Human ZNF541	TGGTTCCAGTGACCCGACACAT	CTTCTTCCGCTTTCTCTGCTGG
Human ZNF747	CCAGGAGTCGGAGGCAGCAAG	GTCTTTCCTCTTCCTTGTTTCTGG
EBV-Z (BZLF1)	CCCAAACTCGACTTCTGAAGATGTA	TGATAGACTCTGGTAGCTTGGTCAA
EBV BMRF1	GAGGAACGAGCAGATGATTGG	TGCCCACTTCTGCAACGA

### VEH | SIV but not THC | SIV BG ECs promote production of infectious HIV

**Fig. 2:**
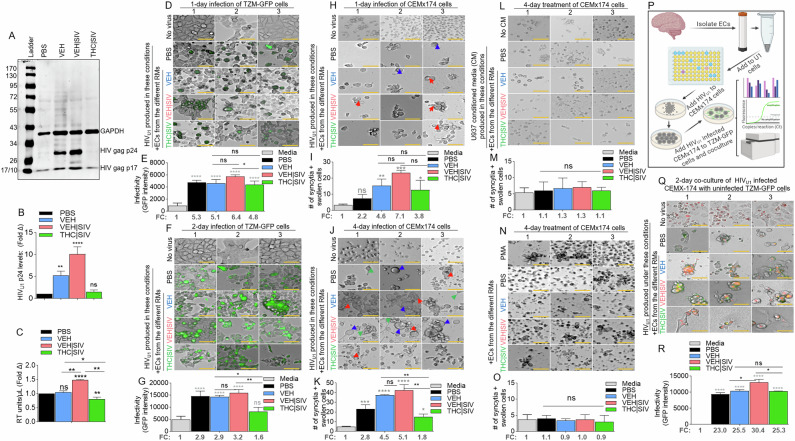
Potency of HIV latency reversal by VEH < VEH | SIV ECs and THC | SIV ECs. **A**) Representative western blot image of intracellular expression of HIV antigens – p24 and p17 in U1 cells, with host (U1 cells) GAPDH used as loading control. **B**) The levels of HIV gag p24 expression (shown in panel A) analyzed by densitometry (ImageJ) and presented as fold change (treatment/PBS). **C**) The level of extracellular HIV RT released by EC-treated U1 cells into the culture supernatants after 4 days of treatment. **D-G**) Clarified U1 cell supernatants collected on day 4 of treatment were added to the indicator cells – TZM-GFP cells and cultured for (**D, E**) one day or (**F, G**) two days. **D, F**) Representative microscopic images of HIV infection (GFP expression, green) after 1 and 2 days of infection respectively. **E, G**) Quantification of HIV infection (GFP expression, green) after 1 and 2 days of infection respectively. The numbers below the bars indicate fold change (treatment/PBS). “No virus” panel shows the level of autofluorescence detected by the instrument. **H-K**) The same clarified U1 cell supernatants collected on day 4 of treatment were added to CEMx174 cells and cultured for (**H, I**) one day or (**J, K**) four days. **H, J**) Representative microscopic images of cell morphology. The number of syncytia and large cells were quantified on **I**) day 1 and **K**) day 4. Red, blue, and green arrows denote syncytia, large cells, and filopodia-like protrusions respectively. **L, M**) Clarified supernatants collected on day 4 from EC-treated U937 cells were added to CEMx174 cells and cultured for 4 days. **L**) Representative microscopic images of cell morphology. **M**) The number of syncytia and large cells were quantified. **N, O**) CEMx174 cells were treated with VEH, VEH | SIV, THC | SIV ECs for 4 days. No virus = cells treated with PBS but not media from EC-treated U1 cells. No CM = cells treated with regular media but not conditioned media. **N**) Microscopic image of cell morphology. **M**) The number of syncytia and large cells were quantified. **P**) Schematic of HIV transfer assay from CEMx174 cells infected with EC-induced HIV cocultured with uninfected indicator TZM-GFP cells. **Q**) Representative microscopic images of HIV transfer assay from CEMx174 (red) to TZM-GFP cells. Green and orange (green + red) indicate infection. **R**) The level of infection (GFP expression). The numbers below the bars indicate fold change (treatment/PBS). All experiments were repeated three times. Statistical differences were assessed by ordinary one-way ANOVA with Tukey’s correction and by Binary Student’s t tests (Welch’s correction). **** p < 0.001, *** p < 0.005, ** p < 0.01, * p < 0.05, and ns = non-significant. Scale bars (horizontal orange lines) = 200 µm.

Compared to PBS and THC | SIV ECs, VEH and VEH | SIV ECs induced significant expression of p24 and p17 in U1 cells (Fig. 2A, B). Cell-free HIV particles from U1 cells treated for 96 h with PBS or ECs were assessed for extracellular reverse transcriptase (exRT) as a proxy for the concentration of cell-free virus [23–25]. While PBS and VEH ECs did not change exRT, THC | SIV ECs significantly decreased but VEH | SIV ECs significantly increased exRT induction (Fig. 2C). HIV particles produced in the presence of VEH | SIV but not VEH and THC | SIV ECs were infectious as shown by GFP expression in TZM-GFP indicator cells on days 1 (Fig. 2D, E) and 2 (Fig. 2F, G).

### VEH | SIV but not THC | SIV BG ECs promote HIV-induced syncytia formation

Compared to CEMx174 cells treated with PBS or THC | SIV ECs, higher levels of cell-cell fusion were observed in cells treated with VEH | SIV > VEH ECs on days 1 (Fig. 2H, I) and 4 (Fig. 2J, K). The appearance of other morphological changes, including swollen cells (Fig. 2J**, blue arrows**) and filopodia-like protrusions (Fig. 2J**, green arrows**) were observed. Interestingly, supernatants from HIV-negative U937 cells (parental cells of U1) treated with PBS or ECs did not induce syncytia (Fig. 2L, M) and direct addition of PMA or any of the ECs to CEM x174 cells did not induce syncytia formation (Fig. 2N, O). These data indicate that syncytia promoting ability of ECs requires the presence of HIV.

### VEH | SIV but not THC | SIV BG ECs potentiate cell-to-cell HIV transfer

Coculture of HIV infected CEM x174 cells with uninfected TZM-GFP cells (Fig. 2P) resulted in virus transfer to the TZM-GFP cells. The level of virus transfer depends on the source of ECs used to reactivate U1 virus for infection. Thus, cells infected with virus from VEH | SIV ECs mediated the highest level of HIV transfer (Fig. 2Q, R). GFP expression in uninfected TZM-bl cells cocultured with infected CEM x174 cells suggest that the EC-reactivated latent HIV virus is fully replicative, and virus dissemination may have occurred through cell-cell contact, with the potential to mediate successful establishment of HIV in atypical cells.

### Durability of BG ECs as latency regulating agents

**Fig. 3:**
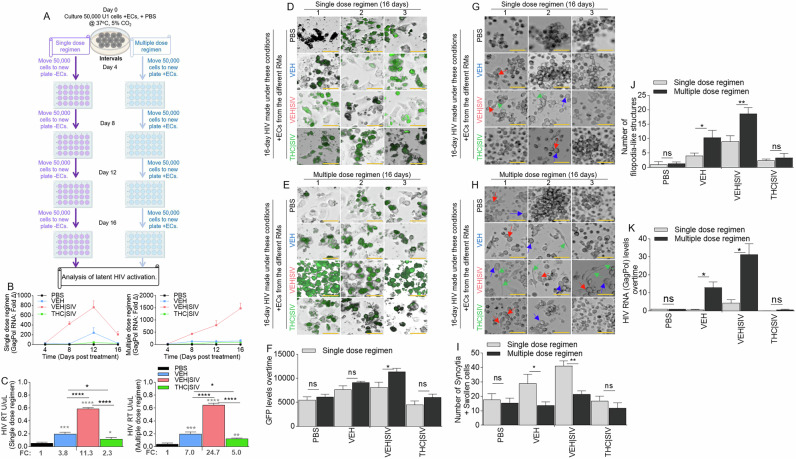
Basal ganglia derived ECs-mediated activation or inhibition of latent HIV reactivation is durable. **A**) Schematic of single and multiple dose regimens of ECs indicating the number of cells, the time of cell passage, the time of EC treatment, and the length of cell culture. **B**) RT-qPCR analysis of the levels of intracellular HIV Gag-Pol expression for single  dose regimen of ECs (left) and multiple dose regimen of ECs (right) of U1 cells. **C**) The level of extracellular HIV RT released by EC-treated U1 cells into the culture supernatants after single dose regimen of ECs (left) and multiple dose regimen of ECs (right). The numbers below the bars indicate fold change (ECs/PBS). **D-F**) Representative microscopic images of HIV infection (GFP expression, green) after **D**) single dose regimen of ECs and **E**) multiple dose regimen of ECs. **F**) Quantification of HIV infection (GFP expression, green) for single and multiple dose regimens of ECs. **G, H**) Representative microscopic images of cell morphology for **G**) single dose regimen of ECs and **H**) multiple dose regimen of ECs. Red, blue, and green arrows denote syncytia, large cells, and cytoplasmic extension respectively. **I**) The number of syncytia and large cells. **J**) The number of filopodia-like structures. **K**) RT-qPCR analysis of the levels of intracellular HIV Gag-Pol expression in CEMx174 cells treated with supernatants from U1 cells subjected to single and multiple dose regimens of ECs. All experiments were repeated three times. Statistical differences were assessed by ordinary one-way ANOVA with Tukey’s correction and by Binary Student’s t tests (Welch’s correction). **** p < 0.001, *** p < 0.005, ** p < 0.01, * p < 0.05, and ns = non-significant. Statistical differences for Fig. 3B were assessed by ordinary 2-way ANOVA with Tukey’s multiple comparison test as presented on Table 3. Scale bars (horizontal orange lines) = 200 µm.

U1 cells treated with single or multiple dose regimen of PBS or ECs (Fig. 3A) over time express HIV RNA mirroring the ECs. HIV RNA in cells treated with a single dose regimen of ECs returned to near baseline on day 16 (Fig. 3B, **left**, Table 3) in contrast with cells treated with multiple dose regimen of ECs where HIV RNA continued to increase up to day 16 (Fig. 3B**, right**, Table 3). The trend of HIV RNA increase was VEH | SIV > VEH > THC | SIV = PBS. Analysis of exRT on day 16 of single or multiple dose regimen of ECs showed similar trends with HIV RNA (Fig. 3C). Importantly, multiple dose regimen of ECs doubled the amount of exRT (11.3 vs 24.7) in culture supernatant compared to single dose regimen (Fig. 3C). Also, multiple dose regimen of ECs resulted in increased GFP expression in TZM-GFP cells compared to single dose regimen (Fig. 3D-F). HIV infectivity was confirmed by syncytia in recipient CEMx174 cells. The number of syncytia and swollen cells were significantly higher in VEH | SIV > VEH cells that received single dose regimen of ECs (Fig. 3G, I) compared to multiple dose regimen of ECs given every 4 days (Fig. 3H, I). THC | SIV did not increase the number of syncytia compared to PBS (Fig. 3G-I). Interestingly, the number of filopodia-like structures were significantly higher in VEH | SIV > VEH cells that received multiple dose regimen of ECs (Fig. 3H, J) compared to single dose regimen of ECs (Fig. 3G, J). The effects of ECs on the release of infectious virus were confirmed by analysis of viral RNA in recipient CEMx174 cells, where ECs induced significant HIV GagPol expression in the following order VEH | SIV > VEH > THC | SIV = PBS (Fig. 3K).

**Table 3:** Statistics for Fig. 3B.

Single dose regimen of ECs			Multiple dose regimen of ECs		
2-way ANOVA (Tukey’s multiple comparisons)	Summary	Adjusted P Value	2-way ANOVA (Tukey’s multiple comparisons)	Summary	Adjusted P Value
Day 1			Day 1		
PBS vs. VEH ECs	**	0.0023	PBS vs. VEH ECs	**	0.0023
PBS vs. VEH | SIV ECs	*	0.0421	PBS vs. VEH | SIV ECs	*	0.0421
PBS vs. THC | SIV ECs	*	0.0144	PBS vs. THC | SIV ECs	*	0.0144
VEH vs. VEH | SIV ECs	ns	0.5220	VEH vs. VEH | SIV ECs	ns	0.5220
VEH vs. THC | SIV ECs	**	0.0013	VEH vs. THC | SIV ECs	**	0.0013
VEH | SIV vs. THC | SIV ECs	ns	0.0584	VEH | SIV vs. THC | SIV ECs	ns	0.0584
Day 2			Day 2		
PBS vs. VEH ECs	ns	0.0581	PBS vs. VEH ECs	*	0.0153
PBS vs. VEH | SIV ECs	*	0.0275	PBS vs. VEH | SIV ECs	**	0.0076
PBS vs. THC | SIV ECs	*	0.0360	PBS vs. THC | SIV ECs	**	0.0082
VEH vs. VEH | SIV ECs	*	0.0292	VEH ECs vs. VEH | SIV ECs	**	0.0060
VEH vs. THC | SIV ECs	ns	0.0707	VEH ECs vs. THC | SIV ECs	*	0.0234
VEH | SIV vs. THC | SIV ECs	*	0.0277	VEH | SIV ECs vs. THC | SIV ECs	**	0.0085
Day 3			Day 3		
PBS vs. VEH ECs	ns	0.3302	PBS vs. VEH ECs	ns	0.3795
PBS vs. VEH | SIV ECs	ns	0.0717	PBS vs. VEH | SIV ECs	*	0.0163
PBS vs. THC | SIV ECs	ns	0.3849	PBS vs. THC | SIV ECs	ns	0.1463
VEH vs. VEH | SIV ECs	ns	0.1199	VEH ECs vs. VEH | SIVECs	**	0.0047
VEH vs. THC | SIV ECs	ns	0.4252	VEH ECs vs. THC | SIV ECs	ns	0.7670
VEH | SIV ECs vs. THC | SIV ECs	ns	0.0754	VEH | SIV ECs vs. THC | SIV ECs	*	0.0150
Day 4			Day 4		
PBS vs. VEH ECs	*	0.0366	PBS vs. VEH ECs>	ns	0.2767
PBS vs. VEH | SIV ECs	ns	0.1937	PBS vs. VEH | SIV ECs	ns	0.0517
PBS vs. THC | SIV ECs	**	0.0052	PBS vs. THC | SIV ECs	***	0.0006
VEH ECs vs. VEH | SIV ECs	ns	0.2376	VEH ECs vs. VEH | SIV ECs	ns	0.0527
VEH ECs vs. THC | SIV ECs	ns	0.7664	VEH ECs vs. THC | SIV ECs	ns	0.9353
VEH | SIV ECs vs. THC | SIV ECs	ns	0.2315	VEH | SIV ECs vs. THC | SIV ECs	ns	0.0604

### VEH | SIV BG ECs reactivate latent HIV in microglia

**Fig. 4:**
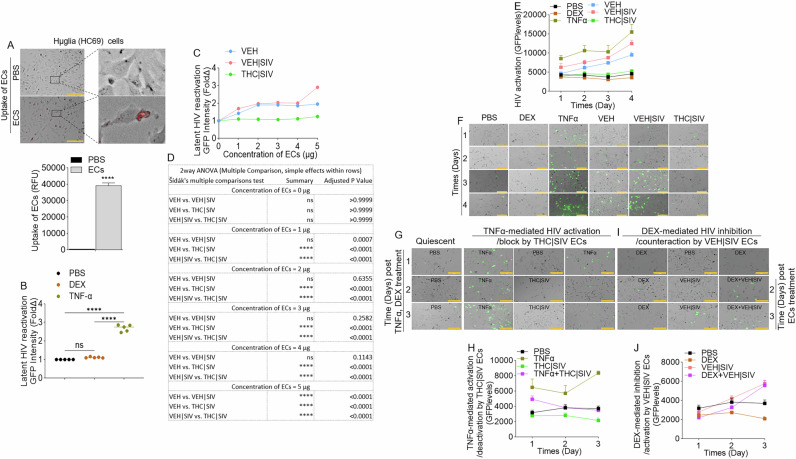
Effect of basal ganglia derived ECs on HIV reactivation in microglia (huglia, HC69) cells. **A**) Representative microscopic images of internalized DIR-labeled ECs by huglia cells (top) and the level of internalized ECs (bars, bottom). Black squares represent enraged images linked with broken lines. **B**) The levels of latent HIV LTR promoter transactivation (GFP expression) by control molecules, including PBS, dexamethasone (DEX) and TNFα). **C**) Concentration (1 µg, 2 µg, 3 µg, 4 µg, 5 µg in 100 µL)-dependent assessment of the potency of ECs from different sources in transactivation of latent HIV LTR promoter at 96 h after treatment. **D**) 2-way ANOVA (Šídák’s Multiple Comparison, simple effects within rows) analysis for the data in panel **“C”**. **E**) Quantification of latent HIV LTR promoter transactivation (GFP expression, green) at different times (days 1, 2, 3, 4) after treatment with VEH, VEH | SIV, THC | SIV ECs and other compounds (PBS, DEX, TNFα). **F**) Representative microscopic images of huglia cells on day 4 after treatment with VEH, VEH | SIV, THC | SIV ECs and other compounds (PBS, DEX, TNFα) showing latent HIV LTR promoter transactivation (GFP expression, green). **G**) Representative microscopic images of hμglia cells showing THC | SIV ECs suppressing TNFα-mediated HIV LTR promoter reactivation. **H**) The levels of HIV reactivation (GFP expression) by TNFα and suppression by THC | SIV ECs. **I**) Representative microscopic images of hμglia cells showing VEH | SIV ECs reversing DEX-mediated inhibition of HIV LTR promoter reactivation. **J**) The levels of inhibition of HIV reactivation (GFP expression) by DEX and reactivation by VEH | SIV ECs. All experiments were repeated three times. Statistical differences were assessed by ordinary 2-way ANOVA with Tukey’s multiple comparison test as presented on Tables 4 – 6. ns = non-significant. Scale bars (horizontal orange lines) = 200 µm.

To directly assess the effects of ECs as latency-reversing agents in microglia cells, we used huglia - HC69 cells [26] that express GFP upon activation (Supplemental Fig. 1A). Cells treated with ECs for 24 h internalized ECs (Fig. 4A). Compared to PBS, dexamethasone (DEX) did not transactivate HIV LTR promoter, while TNFα (10 ng/mL) significantly increased HIV LTR promoter activity, on day 4 post treatment (Fig. 4B). To identify the concentration of ECs needed to transactivate latent HIV in huglia cells, we treated the cells with various concentrations (1, 2, 3, 4, 5 µg) of ECs. While the THC | SIV ECs, irrespective of concentration did not have significant effect on HIV LTR promoter transactivation on day 4 post treatment, VEH and VEH | SIV ECs across all concentrations (1, 2, 3, 4, 5 µg in 100 µL) increased HIV LTR promoter activity compared to PBS (Fig. 4C, D). We did not observe differences in LTR promoter activity in cells treated with 1, 2, 3, or 4 µg of VEH vs VEH | SIV ECs (Fig. 4C, D). Interestingly, 5 µg of VEH | SIV ECs significantly increased HIV LTR promoter activity compared to VEH and THC | SIV ECs (Fig. 4C, D). Moreover, 5 µg VEH | SIV ECs reactivated latent HIV in huglia cells similar to the level seen in TNFα (10 ng/mL) treated cells (Fig. 4E, F). Reactivation of latent HIV was progressive and significantly increased from day 1 to day 4 in the order VEH | SIV > VEH (Fig. 4E, F, Table 4). Huglia cells treated with THC | SIV ECs had quiescent phenotype similar to what was observed in PBS or dexamethasone (DEX, 3 mM/mL) treated cells (Fig. 4B, E–F).

**Table 4:** Statistics for Fig. 4C.

2-way ANOVA (Tukey’s multiple comparisons)	Summary	Adjusted P Value	2-way ANOVA (Tukey’s multiple comparisons)	Summary	Adjusted P Value
**Day 1**			**Day 2**		
PBS vs. DEX	ns	0.4925	PBS vs. DEX	ns	0.7456
PBS vs. TNFα	*	0.0233	PBS vs. TNFα	*	0.0287
PBS vs. VEH ECs	ns	0.8943	PBS vs. VEH ECs	ns	0.2834
PBS vs. VEH | SIV ECs	ns	0.2488	PBS vs. VEH | SIV ECs	*	0.0114
PBS vs. THC | SIV ECs	ns	0.6960	PBS vs. THC | SIV ECs	ns	0.9575
DEX vs. TNFα	*	0.0227	DEX vs. TNFα	*	0.0163
DEX vs. VEH ECs	ns	0.2514	DEX vs. VEH ECs	ns	0.1456
DEX vs. VEH | SIV ECs	ns	0.1624	DEX vs. VEH | SIV ECs	*	0.0108
DEX vs. THC | SIV ECs	ns	0.9178	DEX vs. THC | SIV ECs	ns	0.4812
TNFα vs. VEH ECs	*	0.0248	TNFα vs. VEH ECs	ns	0.0600
TNFα vs. VEH | SIV ECs	ns	0.2063	TNFα vs. VEH | SIV ECs	ns	0.1585
TNFα vs. THC | SIV ECs	*	0.0324	TNFα vs. THC | SIV ECs	*	0.0292
VEH ECs vs. VEH | SIV ECs	ns	0.3528	VEH ECs vs. VEH | SIV ECs	ns	0.5047
VEH ECs vs. THC | SIV ECs	ns	0.3632	VEH ECs vs. THC | SIV ECs	ns	0.4295
VEH | SIV ECs vs. THC | SIV ECs	ns	0.1946	VEH | SIV ECs vs. THC | SIV ECs	*	0.0233
**Day 3**			**Day 4**		
PBS vs. DEX	ns	0.4134	PBS vs. DEX	ns	0.2990
PBS vs. TNFα	ns	0.0645	PBS vs. TNFα	*	0.0318
PBS vs. VEH ECs	**	0.0034	PBS vs. VEH ECs	**	0.0061
PBS vs. VEH | SIV ECs	**	0.0035	PBS vs. VEH | SIV ECs	**	0.0038
PBS vs. THC | SIV ECs	ns	0.3623	PBS vs. THC | SIV ECs	ns	0.3955
DEX vs. TNFα	ns	0.0605	DEX vs. TNFα	*	0.0271
DEX vs. VEH ECs	***	0.0004	DEX vs. VEH ECs	**	0.0036
DEX vs. VEH | SIV ECs	****	<0.0001	DEX vs. VEH | SIV ECs	**	0.0030
DEX vs. THC | SIV ECs	*	0.0251	DEX vs. THC | SIV ECs	*	0.0424
TNFα vs. VEH ECs	ns	0.3233	TNFα vs. VEH ECs	ns	0.1021
TNFα vs. VEH | SIV ECs	ns	0.6995	TNFα vs. VEH | SIV ECs	ns	0.3892
TNFα vs. THC | SIV ECs	ns	0.0951	TNFα vs. THC | SIV ECs	*	0.0420
VEH ECs vs. VEH | SIV ECs	*	0.0271	VEH ECs vs. VEH | SIV ECs	ns	0.0666
VEH ECs vs. THC | SIV ECs	**	0.0090	VEH ECs vs. THC | SIV ECs	*	0.0178
VEH | SIV ECs vs. THC | SIV ECs	***	0.0002	VEH | SIV ECs vs. THC | SIV ECs	*	0.0102

### THC | SIV ECs restrict the reactivation of latent HIV in microglia

Here, we assessed the effects of ECs on TNFα-mediated HIV reactivation and DEX-mediated HIV inhibition in microglia. Latently infected huglia cells were treated with PBS, THC | SIV, VEH | SIV, or stimulated with TNFα (10 ng/mL), Dex (3 mM/mL). Cells were cultured for 1 day. On day 2, THC | SIV ECs were added to TNFα-treated cells while VEH | SIV ECs were added to DEX-treated cells. Reactivation of latent proviruses was evaluated at different time points after treatment (days 1, 2, 3) by measuring GFP expression. Strong inhibition of HIV proviral reactivation in TNFα-stimulated cells was observed in cells treated with THC | SIV ECs treatment (Fig. 4G, H, Table 5). In contrast, strong HIV proviral reactivation in DEX-suppressed cells was observed in cells treated with VEH | SIV ECs (Fig. 4I, J, Table 6). The levels of GFP expression in TNFα + THC | SIV ECs were similar to THC | SIV ECs treated cells and significantly lower than TNFα treated cells (Fig. 4G, H, Table 5), indicating that THC | SIV ECs suppressed the ability of TNFα to reactivate HIV. In parallel, the levels of GFP expression in DEX + VEH | SIV ECs is similar to VEH | SIV ECs treated cells and significantly higher than DEX treated cells (Fig. 4I, J, Table 6), indicating that VEH | SIV ECs countered the ability of DEX to suppress latent HIV activation.

**Table 5:** Statistics for TNFα-mediated activation /deactivation by THC | SIV ECs (GFP levels).

2-way ANOVA (Tukey’s multiple comparisons)	Summary	Adjusted P Value
**Day 1**		
PBS vs. TNFα	ns	0.0694
PBS vs. TNFα + THC | SIV ECs	*	0.0225
PBS vs. THC | SIV ECs	ns	0.4505
TNFα vs. TNFα + THC | SIV ECs	ns	0.2961
TNFα vs. THC | SIV ECs	ns	0.0590
TNFα + THC | SIV vs. THC | SIV ECs	*	0.0138
**Day 2**		
PBS vs. TNFα	ns	0.1818
PBS vs. TNFα + THC | SIV ECs	ns	>0.9999
PBS vs. THC | SIV ECs	ns	0.0977
TNFα vs. TNFα + THC | SIV ECs	ns	0.1892
TNFα vs. THC | SIV ECs	ns	0.0787
TNFα + THC | SIV vs. THC | SIV ECs	ns	0.0531
**Day 3**		
PBS vs. TNFα	***	0.0004
PBS vs. TNFα + THC | SIV ECs	ns	0.9267
PBS vs. THC | SIV ECs	*	0.0192
TNFα vs. TNFα + THC | SIV ECs	**	0.0015
TNFα vs. THC | SIV ECs	***	0.0001
TNFα + THC | SIV vs. THC | SIV ECs	ns	0.0635

**Table 6:** Statistics for DEX-mediated inhibition /activation by VEH | SIV ECs (GFP levels).

2-way ANOVA (Tukey’s multiple comparisons)	Summary	Adjusted P Value
**Day 1**		
PBS vs. DEX	ns	0.1398
PBS vs. DEX + VEH | SIV ECs	ns	0.4281
PBS vs. HIV | SIV ECs	ns	0.4505
DEX vs. DEX + VEH | SIV ECs	ns	0.9492
DEX vs. HIV | SIV ECs	ns	0.3500
DEX + VEH | SIV vs. HIV | SIV ECs	ns	0.7146
**Day 2**		
PBS vs. DEX	ns	0.0881
PBS vs. DEX + VEH | SIV ECs	ns	0.3475
PBS vs. HIV | SIV ECs	ns	0.5759
DEX vs. DEX + VEH | SIV ECs	ns	0.1253
DEX vs. HIV | SIV ECs	**	0.0097
DEX + VEH | SIV vs. HIV | SIV ECs	*	0.0381
**Day 3**		
PBS vs. DEX	*	0.0155
PBS vs. DEX + VEH | SIV ECs	*	0.0313
PBS vs. HIV | SIV ECs	**	0.0067
DEX vs. DEX + VEH | SIV ECs	**	0.0082
DEX vs. HIV | SIV ECs	***	0.0009
DEX + VEH | SIV vs. HIV | SIV ECs	ns	0.9408

### ECs reprogram transcriptome of HIV latently infected monocytes

**Fig. 5:**
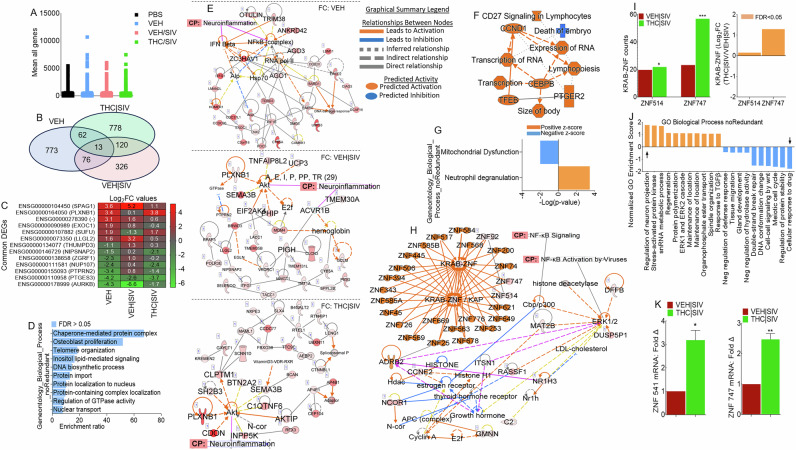
Global transcriptome analysis of HIV latently infected U1 cells treated with basal ganglia derived ECs. **A**) Scatter plot of all transcripts from biological triplicates with transcripts per million mapped reads (TPM) > 1, p-value (unpaired t-test) <= 1.0, and fold-change >= 0.0 are displayed. **B**) 3-way Venn diagram of DEGs meeting the following criteria: i) pooled-adjusted p-value (FDR) < 0.05 and ii) (log_2_ fold change >1 or <-1). **C**) Heatmap of the genes corresponding to the 13 common DEGs identified by the 3-way Venn diagram. **D**) Overrepresentation analysis of gene ontology biological processes of the 13 common DEG identified by the 3-way Venn diagram. The y-axis represents biological process (noRedundant) and the x-axis represents enrichment ratios. **E**) IPA network diagrams representing the regulatory effects with top consistency scores found associated with neuroinflammation signaling, with the top network representing FC: VEH  ECs/PBS, middle network representing FC: VEH | SIV  ECs/PBS, and bottom network representing FC: THC | SIV  ECs/PBS. FC = Fold Change. **F**) Gene to function analysis of THC | SIV  ECs/VEH | SIV  ECs DEGs. **G**) Histogram of the 92 canonical pathways regulated by treatment with THC | SIV ECs. The left y-axis represents the canonical pathways, and the x-axis represents -log (P-value) of the canonical pathways, where neutrophil degranulation is upregulated, and mitochondrial dysfunction is downregulated. Each bar indicates the value of -log_10_ (P-value). **H**) IPA network diagram representing top consistency scores with links to NF-kB activation by viruses and signaling, as well as KRAB-ZNF (Krüppel-associated box domain zinc finger) gene cluster. **I**) Differences in ZNF514 and ZNF747 represented as counts (left) and log_2_ fold change (right). **J**) Gene ontology gene set enrichment analysis (GSEA). The left y-axis represents normalized GO enrichment scores, and the x-axis represents GO biological process (noRedundant). **K**) DEGs validated with RT-qPCR analysis. Statistical differences were assessed by ordinary one-way ANOVA with Tukey’s correction and by Binary Student’s t tests (Welch’s correction). **** p < 0.001, *** p < 0.005, ** p < 0.01, * p < 0.05, and ns = non-significant.

We mapped U1 RNA-seq reads to RefSeq-annotated human gene loci, quantified reads as transcripts per million mapped reads (TPM) and genes are considered expressed if TPM > 1 in all three samples within a treatment group. Data filtered with p-value (unpaired t-test) <= 1.0 and FC > = 0.0, resulted in 14440 mRNAs (Fig. 5A, Supplemental Table 1). Differentially expressed genes (DEGs) identified using p-value (unpaired t-test) <0.05, p-adj (FDR < 0.05) were 898, 507, and 943 for VEH  ECs, VEH | SIV  ECs, and THC | SIV respectively (Supplemental Table 2). 3-way Venn overlap analysis identified EC-altered genes that are either unique or common to all 3 treatments (Fig. 5B, Supplemental Table 3). The expression levels of the common genes are shown on the heatmap (Fig. 5C). Of the 13 common genes, VEH | SIV ECs upregulated 8 and down regulated 5 genes while THC | SIV ECs had the opposite effect. Of interest was the upregulation by VEH | SIV ECs and downregulation by THC | SIV ECs of LLGL2 and PTPRN2. LLGL2 forms a complex with GPSM2/LGN, PRKCI/aPKC and PARD6B/Par- 6 to ensure the correct organization and orientation of bipolar spindles for normal cell division. PTPRN2 is a receptor-type tyrosine-protein phosphatase N2 required for normal accumulation of secretory vesicles in the hippocampus, and normal accumulation of neurotransmitters (norepinephrine, dopamine, serotonin) in the brain. Also of interest is the differential effect of ECs on PLXNB1, which was downregulated by VEH | SIV ECs and upregulated by THC | SIV ECs. PLXNB1 is a receptor for SEMA4D, which plays a role in GABAergic synapse development, mediates SEMA4A- and SEMA4D-dependent inhibitory synapse development, and plays a role in RHOA activation and subsequent changes of the actin cytoskeleton, axon guidance, invasive growth and cell migration. Similarly, AURKB was downregulated by VEH | SIV ECs and upregulated by THC | SIV ECs. Interestingly, AURKB is a key cell cycle regulatory kinase known to inhibit HIV cell-to-cell transmission [27], similar to the effect of THC | SIV ECs that inhibits cell-to-cell transmission (Fig. 3Q, R). Overrepresentation analysis of the common genes identified important gene ontology (GO) biological processes associated with the 13 common genes in Fig. 5C, where 11 genes were annotated to the GO biological process and used for the enrichment analysis (Fig. 5D).

### Gene-to-function analysis of the transcriptome of EC-treated cells predicts links to neuroinflammation

IPA pathway and network analyses of genes in Fig. 5B showed that ECs regulate various canonical pathways, biological, cellular, diseases, and molecular functions (Table 7). The different ECs are predicted to regulate biological processes with neurological disease as the first of the top 5 diseases for VEH | SIV  ECs treated cells and the last of the top 5 diseases for THC | SIV  ECs treated cells (Table 7). Treatment with VEH ECs was linked to 11 networks that have 29 focused molecules with activated IFNβ and NF-kB complex as hub networks driving neuroinflammation signaling (Fig. 5E**, top**, Supplemental Fig. 2). VEH | SIV  ECs treated cells had 9 networks with 30 focused molecules with activated Akt and ACVR1B as hub networks driving neuroinflammation signaling (Fig. 5E**, middle**, Supplemental Fig. 3). On the other hand, THC | SIV  ECs treated cells had 20 networks with 30 focused molecules and activated Akt as a hub network driving neuroinflammation signaling (Fig. 5E, **bottom**, Supplemental Fig. 4). Detailed IPA analysis revealed that VEH ECs may regulate neuroinflammation by downregulating NF-kB complex leading to inhibition of TGFβ, neuronal, and microglia survival, and increased accumulation of nitric oxide, while activating IFNβ signaling, which leads to increased myelin debris clearance and inhibition of activated MHC Class II (Supplemental Fig. 2). Although VEH | SIV  ECs and THC | SIV ECs regulate neuroinflammation via Akt, the Akt-mediated regulation of neuroinflammation function can be achieved through different executioner molecules. VEH | SIV ECs may regulate neuroinflammation via Akt interaction with glycogen synthase kinase 3 beta (GSK3B) mediated inhibition of NF-kB activation that leads to inhibition of TGFβ, inhibition of neuronal and microglia survival, and increased accumulation of nitric oxide, inhibition of activated MHC Class II, as well as GSK3B mediated blockade of AP1 (Supplemental Fig. 3). Unlike VEH | SIV ECs, THC | SIV ECs mediate neuroinflammation via Akt interaction with GSK3B mediated activation of NF-kB signaling that leads to the activation of TGFβ, increased neuronal and microglia survival, and decreased accumulation of nitric oxide and inhibition of MHC Class II, as well as GSK3B mediated blockade of activated AP1 (Supplemental Fig. 4). These findings are significant because Akt-induced NF-kB via GSK3B may promote or impair survival of latently infected cells.

**Table 7:** Three Biological Processes induced by different treatments with ECs.

Group	Top 5 Canonical Pathways	
	Name	p-value range
**VEH ECs**	RNA Pol III Transcription	1.25E-04
	Assembly of RNA Pol III Complex	6.19E-03
	mRNA Capping	1.19E-02
	Nucleotide Excision Repair Pathway	1.44E-02
	Nucleosome assembly	2.01E-02
**VEH** | **SIV ECs**	Metabolism of vitamin K	5.29E-03
	PI Metabolism	9.47E-03
	Xanthine and Xanthosine Salvage	1.06E-02
	Cell Cycle Checkpoints	1.50E-02
	Guanine and Guanosine Salvage	1.58E-02
**THC** | **SIV ECs**	PI Metabolism	2.49E-03
	Mitotic Roles of Polo-Like Kinase	7.25E-03
	Cilium Assembly	8.51E-03
	NAD Phosphorylation and Dephosphorylation	9.32E-03
	Gamma carboxylation, hypusine formation and arylsulfatase activation	1.14E-02
	**Top 5 Diseases and Biological Functions**	
**VEH ECs**	**Name**	**p-value range**
	Connective Tissue Disorders	4.14E-04 - 4.14E-04
	Developmental Disorder	4.14E-04 - 4.14E-04
	Hereditary Disorder	2.21E-02 - 4.14E-04
	Organismal Injury and Abnormalities	4.80E-02 - 4.14E-04
	Skeletal and Muscular Disorders	2.13E-02 - 4.14E-04
**VEH** | **SIV ECs**	Neurological Disease	4.92E-02 - 5.77E-07
	Organismal Injury and Abnormalities	4.92E-02 - 5.77E-07
	Cancer	4.92E-02 - 3.41E-06
	Dermatological Diseases and Conditions	4.67E-02 - 5.75E-04
	Gastrointestinal Disease	4.67E-02 - 9.10E-04
**THC** | **SIV ECs**	Cancer	4.49E-02 - 7.36E-17
	Organismal Injury and Abnormalities	4.49E-02 - 7.36E-17
	Gastrointestinal Disease	4.27E-02 - 1.10E-13
	Endocrine System Disorders	4.42E-02 - 4.83E-10
	Neurological Disease	4.49E-02 - 1.14E-07
	**Top 5 Molecular and Cellular Functions**	
	**Name**	**p-value range**
**VEH ECs**	Cell Death and Survival	3.54E-02 - 4.14E-04
	Cellular Assembly & Organization	2.74E-02 - 4.14E-04
	DNA Replication, Recombination, Repair	2.13E-02 - 4.14E-04
	Small Molecule Biochemistry	2.13E-02 - 8.27E-04
	Cell Cycle	2.74E-02 - 1.24E-03
**VEH** | **SIV ECs**	Cell Death and Survival	4.25E-02 - 5.77E-07
	Cell Morphology	4.67E-02 - 5.77E-07
	Cellular Compromise	3.13E-02 - 5.77E-07
	Cellular Assembly and Organization	4.67E-02 - 1.77E-03
	Cellular Function and Maintenance	4.67E-02 - 1.77E-03
**THC** | **SIV ECs**	Cell Morphology	4.49E-02 - 1.30E-04
	Cell Death and Survival	4.49E-02 - 6.51E-04
	Cellular Assembly and Organization	4.49E-02 - 1.10E-03
	Cellular Function and Maintenance	4.49E-02 - 1.10E-03
	Carbohydrate Metabolism	4.49E-02 - 1.89E-03

### THC | SIV ECs regulate expression of KRAB- ZNF gene family

We identified 745 DEGs that are significantly modified by THC | SIV  ECs (Supplemental Table 2). Gene-to-function analysis identified 9 upregulated and 1 downregulated biological process (Fig. 5F). Neutrophil degranulation and mitochondrial dysfunction are the most upregulated and downregulated pathways, respectively (Fig. 5G, Supplemental Fig. 5, **black arrows**). A total of 19 IPA biological networks were identified. Merging of networks 8 and 17 linked to neurological disease identified KRAB-ZNF cluster containing 24 family members and linked to ERK1/2 activation and NFkB activation via ADRB2 (Fig. 5H), a regulator of the neuroimmune response [28]. Out of the 24 ZNF family members, ZNF514 and ZNF747 were present in our data set, and both were upregulated by THC | SIV ECs (Fig. 5I). GO gene set enrichment analysis (GSEA) of KRAB- ZNF cluster predicted THC | SIV  ECs treatment to be associated with GO terms that included increased regulation of neuron projection development and decreased cellular response to drugs among others (Fig. 5J). ZNF514 and ZNF747 expression (Fig. 5K) were validated with RT-qPCR using primer sequences in Table 2.

### THC | SIV ECs augment secretion of TH2 and suppression of inflammasome activating cytokines

**Fig. 6:**
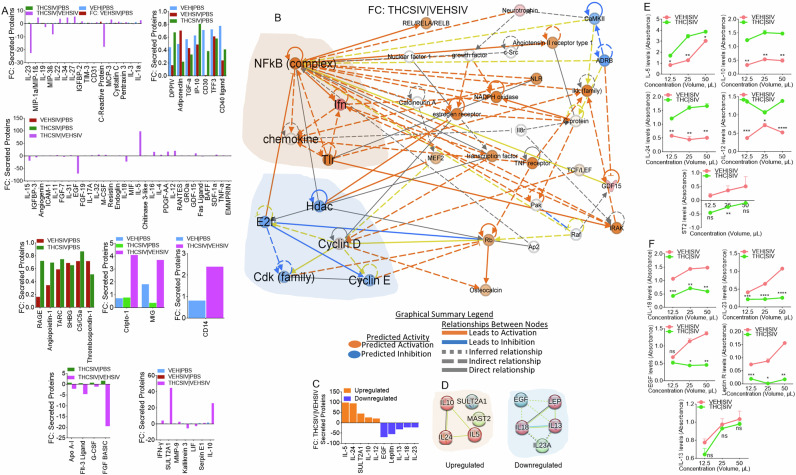
Altered secretion of proteins by HIV latently infected U1 cells treated with basal ganglia derived ECs. **A**) Multiple plots of the fold change differences amongst all the common proteins are plotted as bar graphs. The y-axis represents Fold Change, and the x-axis represents significantly enriched proteins. **B**) IPA network diagram representing the regulatory networks with top consistency scores found associations with different molecules. **C**) Levels of top 10 differentially secreted proteins between THC | SIV ECs/VEH | SIV ECs. The y-axis represents Fold Change, and the x-axis represents differential proteins. **D**) STRING interactome analysis of upregulated (left) and downregulated (right) proteins. The blue, green, and red nodes represent different clusters. The line thickness indicates the strengths of the interactions. **E, F**) The preferential secretion of the proteins in panel “**C**” was validated by ELISA analysis. Five (IL-5, IL-10, IL-24, IL-12, ST2) out of the five upregulated proteins were validated (**E**). Four (IL-18, EGF, IL-23, Leptin) out of the five downregulated proteins were validated, while there was no difference in the levels of IL-13 in the ELISA assay (**F**).

Secretome analysis of supernatants from U1 cells treated with PBS or ECs identified 105 secreted proteins (Supplemental Fig. 6A). 34, 91, 68, and 81 proteins were significantly altered in supernatants from treated with VEH ECs, VEH | SIV ECs, THC | SIV ECs, and THC | SIV ECs/VEH | SIV ECs (Supplemental Fig. 6B). A 4-way Venn overlap analysis identified proteins that are common amongst groups or those that are unique to each group (Supplemental Fig. 6C). The fold change differences amongst all the common proteins are displayed as bar graphs (Fig. 6A). Network analyses of significantly different proteins identified the main hub proteins of each network (Fig. 6B, Supplemental Fig. 7). The levels (Fig. 6C) and PPI network of the top 5 upregulated (Fig. 6D**, left**) and top 5 downregulated (Fig. 6D**, right**) proteins were determined. Upregulated proteins are in 3 clusters with the Th2 cytokines IL-5, IL-10, IL-24 in a cluster linked to the GO biological process related to regulation of signaling pathway via JAK-STAT. Downregulated proteins are in 3 clusters with IL-13, IL-18, LEP in a cluster linked to GO biological processes related to regulation of natural killer cell proliferation and inflammatory bowel disease in KEGG pathway. Secretion of all five (IL-5, IL-10, IL-24, IL-12, sST2) proteins were confirmed by ELISA assay using supernatants of huglia cells treated with VEH | SIV ECs and THC | SIV ECs (Fig. 6E). In parallel, IL-18, IL-23, Leptin, EGF, but not IL-13 was validated by ELISA using the same supernatants (Fig. 6F).

### VEH | SIV ECs but not THC | SIV ECs reversed HIV latency in primary PBMC from HIV+ individuals treated with ART

Thus far, we have shown that ECs regulate reversal of latent HIV in cell line models of HIV latency. Here, we assessed HIV latency reversal capability of ECs on primary PBMC from HIV+ individuals on stable ART for 1 to 13 years (Table 8).

**Table 8:** General characteristics of HIV-infected (HIV + ) ART-suppressed (HIV + ART + ) PBMC donors.

Donors	Age	Sex	Race	Viral load (copies/mL)	CD4 T cell count (cells/µL)	ART regimen	Years on ART
1	36	M	C	ND	840	Triumeq (abacavir, 3TC, dolutegravir)	11.75
2	48	M	AA	< 50	508	Biktarvy (TAF, FTC, bictegravir)	6
3	34	M	B	ND	1294	Odefsey (TAF, FTC, rilpivirine)	13
4	36	F	B	< 50	281	Biktarvy (TAF, FTC, bictegravir)	1

*ND* none detected, AA african american *B* black, *C* caucasians.

**Fig. 7:**
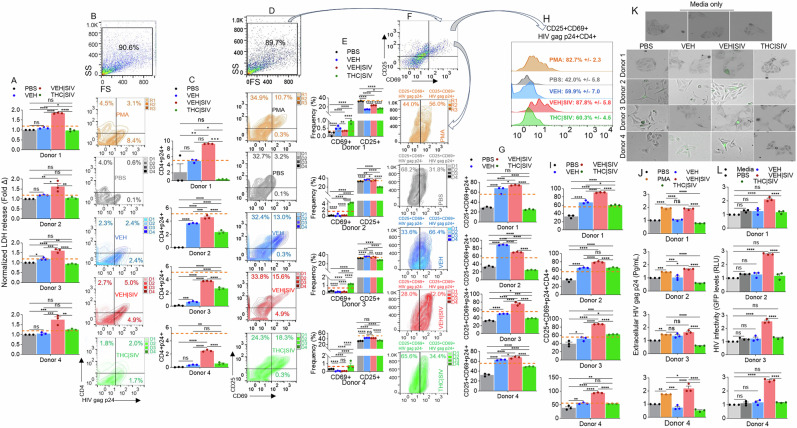
VEH | SIV ECs reactivated the expression of HIV gag p24 in primary PBMC from HIV+ individuals treated with ART. **A**) Levels of LDH released by PBMCs from all donors treated with ECs and PMA compared to PBS treated PBMCs. **B**) Flow cytometry analysis showing plots of gate of side scatter (SS) and forward scatter (FS) and their associated zebra plot quadrants for CD4 and HIV gag p24. **C**) Frequencies of CD4+ and HIV gag p24+ cells for all donors. **D**) Flow cytometry analysis showing plots of gate of side scatter (SS) and forward scatter (FS) and their associated zebra plot quadrants for CD25 and CD69. **E**) Frequencies of CD69+ and CD25+ cells for all donors. **F**) Flow cytometry analysis showing plots of gate of side scatter (SS) and forward scatter (FS) and their associated zebra plot for CD25 + CD69+ (arrows) for CD25 + CD69 + HIV gag p24- and CD25 + CD69 + HIV gag p24 + . **G**) Frequencies of CD25 + CD69 + HIV gag p24+ cells for all donors. **H**) Histogram shows plots of gate of CD25 + CD69 + HIV gag p24CD4+ and the mean frequencies +/- standard deviation for four donors. **I**) Frequencies of CD25 + CD69 + HIV gag p24 + CD4+ cells for all donors. **J**) Levels of extracellular HIV gag p24 (Pg/mL) measured in PBMCs culture using p24 ELISA. **K**) Representative microscopic images of HIV infection (GFP expression, green) after 72 h (3 days) of treatment with supernatants from PBMC cultures from HIV + ART + . **L**) Quantification of HIV infection (GFP expression, green) after 72 h (3 days) of treatment with supernatants from PBMC cultures from HIV + ART + . Where indicated, PMA served as positive control plotted as horizontal lines. PBS represent PBMCs treated with PBS without ECs and serve as no EC control. Media represent TZM-GFP cells treated with culture media alone. Statistical significance was determined by ordinary one-way ANOVA (Šídák’s multiple comparisons test) **** p < 0.0001, *** p < 0.0005, ** p < 0.0088, * p < 0.01, ns = non-significant.

Recruitment characteristics were as follows: Donors were at least 18 years old, receiving ART ( ≥ three drugs, ≥ 1 year), with more than 200 CD4 + T cells/μL of blood, with a history of nadir CD4 + T cell count of at least 100 cells/μL, and plasma HIV-1 RNA of ≤50 copies/mL at the time of participation. Cryopreserved PBMCs were rested and treated as described in the methods section following previously published protocol [29]. Treatment with VEH | SIV ECs resulted in significant increases in L-Lactate dehydrogenase (LDH) release by PBMCs from all donors, compared to VEH ECs treated cells (Fig. 7A). VEH | SIV ECs-induced LDH release was higher than PMA-induced LDH release. THC | SIV ECs did not have effect on the levels of secreted LDH (Fig. 7A). These data show that in response to VEH | SIV ECs, but not VEH ECs and THC | SIV ECs exogenously applied to the PBMCs, LDH is released from the cytoplasm into the extracellular environment, suggesting that VEH | SIV ECs may affect PBMC viability. Treatment of PBMCs with ECs led to variable responses in the frequencies of CD4+ cells expressing the HIV antigen p24 gag (Fig. 7B, C). While VEH | SIV ECs consistently led to significant increases in CD4 + HIVp24+ cells in all donor cells, VEH ECs and THC | SIV ECs increased CD4 + HIVp24+ cell frequencies, although the increase is significantly lower than that observed in PBMCs treated with VEH | SIV ECs (Fig. 7B, C).

To link the functional outcome of ECs treatment to host cell activation, we assessed the expression of the activation antigen CD69, which predicts functionality of in vitro expanded PBMCs from HIV-infected patients [30]. While all ECs altered the frequencies of CD69+ cells from all donors, the increase in CD69 expressing cells was more pronounced in cells treated with THC | SIV compared to VEH | SIV ECs treated cells (Fig. 7D, E). Unlike in CD69 expression, CD25 expression is significantly upregulated in donors 2 to 4 following stimulation with VEH ECs and VEH | SIV ECs and downregulated in all donor PBMCs treated with THC | SIV ECs (Fig. 7D, E).

Additionally, treatment with ECs altered the levels of cell-associated HIV gag p24 in CD25 + CD69+ (CD25 + CD69 + HIV p24 + ) PBMCs (Fig. 7D, F, G). In all donor PBMCs, VEH ECs and VEH | SIV ECs increased the frequencies of CD25 + CD69 + HIV gag p24 + , while THC | SIV ECs decreased the frequencies of CD25 + CD69 + HIV p24+ (Fig. 7G). The frequencies of the CD25 + CD69 + HIV gag p24 + CD4+ were also significantly higher in donor PBMCs treated with VEH | SIV ECs > VEH ECs > THC | SIV ECs > PBS (Fig. 7H, I).

Further analysis focused on the extracellular HIV protein was conducted with PBMCs culture supernatant for the release of HIV gag p24 calculated by a p24 ELISA at a 1:200 dilution. The data showed that in line with cell-associated HIV gag p24, the amount of extracellular HIV gag p24 released into the culture supernatants of the PBMCs were altered by treatment with ECs. HIV gag p24 secreted into PBMCs culture is consistently significantly higher in cultures treated with VEH | SIV ECs > THC | SIV ECs (Fig. 7J). These data suggest that ECs play roles in HIV persistence by regulating the size of translational competent HIV reservoirs.

### VEH | SIV ECs-treated PBMCs from HIV+ individuals on ART produce replication competent HIV

Given the detection of significant increase in cell-associated HIV gag p24 expression in PBMCs treated with VEH ECs or VEH | SIV ECs (Fig. 7B, C, F–I) and the release of gag p24 into the PBMC cultures (Fig. 7J), we sought to determine whether this HIV reactivation with VEH ECs or VEH | SIV ECs was associated with the production of infectious virions. Hence, culture supernatants from the PBMCs were added to indicator TZM-GFP cells and cultured for 72 h. Addition of culture supernatants obtained from PBMCs treated with VEH | SIV ECs resulted in induction of GFP expression that is indicative of infection (Fig. 7K). Interestingly, while VEH ECs induced HIV protein (HIV gag p24) expression, it failed to induce GFP expression in the indicator TZM-GFP cells in comparison to cells treated with media alone or culture supernatants from PBS treated PBMCs. A similar observation was made with TZM-GFP cells treated with culture supernatants from PBMCs treated with THC | SIV ECs (Fig. 7K). GFP^+^ TZM-GFP cells were quantified using the Gen5 software of LionHeart. Consistent with the images, GFP expression was significantly upregulated in cells treated with culture supernatants from VEH | SIV ECs-treated PBMCs (Fig. 7L). These data suggest that infectious virions were present in PBMC culture supernatants treated with VEH | SIV ECs and that the virions were capable of mediating cell-free infection. The use of PBMCs is relevant to in vivo situation as CD4 T cells are not present as isolated clusters but circulate as a heterogeneous mixture together with monocytes, other T cells, B cells, eosinophils, basophils, mast cells, etc. in blood. Moreover, the same PBMCs treated with ECs from the different treatment groups yielded results similar to those observed in microglial cells.

### VEH | SIV ECs but not THC | SIV ECs induce Epstein-Barr virus (EBV) lytic gene expression

To determine whether the effects of ECs extend to other latent viruses, we used EBV type I and type III latency cell lines Akata and Raji [31] as prototype EBV latency models. The cells were treated with PBS or the different ECs and the expression of immediate-early viral transactivator, BZLF1 (EBV Z) [32–34] that encode the Z protein and EBV promoter BMRF1 [35, 36], which is an early lytic viral protein that functions as the viral DNA polymerase processivity factor [37] were examined. We found that VEH | SIV ECs induced the expression of BZLF1 (Z) and BMRF1 in contrast to VEH ECs and THC | SIV ECs in Akata (Supplemental Fig. 8A) and Raji (Supplemental Fig. 8B) cell lines. These data indicate that VEH | SIV ECs but not VEH ECs or THC | SIV ECs induced EBV lytic program that can initiate the expression of the viral immediate-early gene BZLF1. Further assessment showed that the concentration of ECs (50 µg/mL) used has variable but subtle effects on cells. While VEH ECs and VEH | SIV ECs did not have effects on Raji cells, THC | SIV ECs decreased the viability of Akata (Supplemental Fig. 8C) and Raji (Supplemental Fig. 8D) cell lines. Although the decrease is significant, it was subtle and not responsible for the induction of the EBV lytic program.

## Discussion

In this study, we showed that BG-derived ECs are previously unknown endogenous latency regulating agents. VEH | SIV ECs potently induced durable activation of latent HIV in three different cell lines (J-Lat GFP/J-Lat Tat-GFP T cells, U1 monocytes, HC69 microglia) and primary T cell models of HIV latency as evidenced by transactivation of HIV LTR promoter, expression of cell-associated HIV mRNA and gag p24, as well as release of infectious virions. In contrast, THC | SIV ECs either did not or minimally reactivated latent HIV in the three cell lines (Figs. 1–4). The reason for the different responses in the levels of HIV gag p24 (Fig. 2A, B) is yet to be determined. The presence of p17 and the absence of p24 in PBS and THC/SIV ECs treated U1 cells may mean that the viral particles are disrupted or incomplete. This may mean that the virus is not fully assembled and may not be infectious. The p17 matrix proteins indicate the presence of the building blocks in cells treated with VEH ECs and THC | SIV ECs, but the capsid (p24) core structure needed for infection is lacking. Additionally, when latently HIV infected cells are cultured, changes in temperature, humidity and oxygen tension can cause varying degrees of cellular activation, which can lead to some activation of the integrated latent HIV genome [38]. This may explain the p17 protein in the PBS-treated cells. However, the data show that only VEH ECs (which have cellular proteins and RNA) and more strongly VEH/SIV ECs (that contains strong proinflammatory cargo) showed mild to strong production of the p24 capsid protein. Noteworthy, PBMCs treated with VEH ECs showed upregulated HIV gag p24 protein expression (Fig. 7C,G), no change in extracellular HIV gag p24 (Fig. 7J), and culture supernatants from these PBMCs were unable to infect TZM-GFP indicator cells (Fig. 7J,K). These observations indicate that infectious viral particles were not significantly produced in such culture conditions.

In U1 cells, virions produced by EC-treated cells induced syncytia formation both in short- and long-term assays and were infectious as they mediated cell-free and cell-to-cell viral spread in the following order VEH | SIV ECs > VEH ECs > THC | SIV ECs = PBS. The induction of syncytium or lack thereof by VEH | SIV ECs or THC | SIV ECs, respectively, are significant because syncytium is a form of cell fusion that likely forms due to excess karyogamy - nuclear fusion within the syncytium [39], where giant cells are formed by infected cells fusing with neighboring uninfected cells, as a means of disseminating the virus [40, 41]. The inhibitory effects of THC | SIV ECs suggest that THC | SIV ECs do not have proinflammatory cargo to activate latently infected cells or that the THC | SIV ECs could induce anti-inflammatory responses and may drive HIV into ‘super latency’. THC | SIV ECs-mediated promotion of latency is durable because it blocked HIV transcription up to 16 days with or without additional treatment (Fig. 3) and failed to reactivate latent HIV despite treatment with potent reactivators like TNFα (Fig. 4D-G).

ECs mediate specific transcriptional profiles and gene networks that regulate neuroinflammation. In the presence of VEH ECs and VEH | SIV ECs, Akt activation may result in loss of NF-kB activation via GSK3B while THC | SIV ECs may result in stimulation of NF-kB by Akt (Supplemental Figs. 2–4). These results demonstrate two separate functions of the Akt complex in NF-kB activation in HIV latently infected cells. Furthermore, THC | SIV EC-mediated activation of KRAB-ZNF gene cluster is remarkable but their contribution to HIV pathogenesis remains unclear. Activation of KRAB-ZNF cluster (Fig. 5H) is interesting because KRAB-ZNF may have pleiotropic effects on transcriptional regulation of its target genes [42] since they bind target promoters via specific DNA recognition sequences and regulate transcription by RNA pol II. In HIV infected cells, intact proviruses preferentially integrate within KRAB–ZNF genes and ZNF genes carrying clonal intact integrations are down-regulated upon cellular activation [43]. ZNF304 silences HIV gene transcription via recruitment to the viral promoter of heterochromatin-inducing methyltransferases [44]. KRAB-ZNF proteins function as potent transcriptional repressors and thus, in addition to the local chromatin environment, KRAB-ZNF proteins may be critical regulators of viral latency. Whether or not ZNF514 and ZNF747 genes regulate HIV latency remains to be determined. Put together, studies described in Fig. 5 revealed how the different ECs altered the transcriptome of HIV latently infected U1 cells and identified predicted transcriptional profiles and gene networks that may regulate neuroinflammation.

Of note, the secretome profile of cells treated with THC | SIV ECs clearly distinguished functions of the ECs in suppressing reactivation of latent proviruses. In the setting of THC | SIV ECs, gene-to-function analyses revealed activated NF-kB complex, which is a hub for IFN, TLR, and chemokine activation that may contribute to the control of virus replication. Causal network analysis of **i**) NF-kB activation by viruses via ERK1/2, and **ii**) NF-kB signaling via Cbp/p300, may mediate gene expression by regulating chromatin structure at the gene promoter through their intrinsic histone acetyltransferase (HAT) activity. Such activities may promote or block the recruitment of basal transcriptional machinery, including RNA pol II, to the HIV promoter. Interestingly, THC | SIV ECs preferentially increased the levels of Th2 cytokines IL-5, IL-10, and IL-24, but decreased the levels of proinflammatory molecules IL-18, IL-23, Leptin, and EGF. Pro-inflammatory properties of leptin are similar to those of the acute phase reactants and upregulates the secretion of inflammatory cytokines [45]. The proinflammatory cytokine IL-18, that is mainly secreted by myeloid cells plays an important role in host response to infection by viruses and intracellular pathogens [46]. IL-18 is elevated in the serum of PWH [47], increased during ART failure but decreased in virally suppressed individuals [48]. THC | SIV ECs-mediated suppression of IL-18 secretion, inhibition of HIV transcription, and production of infectious virions suggest that IL-18 may regulate HIV persistence by enhancing reactivation of latent proviruses and/or increasing HIV replication, as suggested for HIV infected monocytes and T cell lines [49–51]. The current findings support our previously published studies demonstrating that treatment of SIV-infected macaques with THC results in suppressed inflammation and secretion of extracellular vesicles (EVs) with anti-inflammatory functions [9–11, 15, 16, 52].

The ability of latently HIV infected cells to respond to stimulation with VEH ECs but more potently by VEH | SIV ECs suggests that these endogenous ECs can reverse HIV latency in vitro. In the absence of ART or failed treatment, these ECs may provide a trigger for persistent viremia in vivo. On the other hand, the Th2-biased secretome signature (increased secretion of IL-5, IL-10, IL-24) by THC | SIV ECs-treated monocytes and microglia in addition to the inhibition of HIV transcription support the notion that THC | SIV ECs inhibit reactivation of latent HIV, suppress inflammation, and may even block and lock the latent HIV proviral genome from reactivation. While there is no consensus on the role of IL-10 in HIV pathogenesis [53], increased levels of IL-10 was shown to inhibit HIV replication in vitro [54] and elevated plasma levels of IL-10 was associated with the control of viral replication in pregnant women [55]. Although increased levels of IL-10 in PWH may be seen as deleterious due to the potential of IL-10 to decrease the production of Th1 cytokines and skew host response towards Th2 [56], there is broad unanimity in the HIV field that progression of HIV and non-AIDS associated comorbidities is closely linked to persistent immune activation.

Aside from observations of the effects of ECs in regulating latent HIV in various latency cell line models, data presented in Fig. 7 underscore the involvement of ECs in reactivation of latent HIV in primary human PBMCs. The observation that VEH | SIV ECs mediated reactivation of latent HIV in primary PBMC from HIV+ individuals treated with ART is remarkable and indicates that such ECs maybe associated with the production and secretion of replication competent virus, as shown in Fig. 7J, K. The production of infectious HIV by VEH | SIV ECs could potentially contribute to viral rebound in vivo. Although latently infected resting memory CD4 T cells are major HIV reservoir carrying integrated viral genome that does not produce virions [57, 58], a suggested approach for the eradication of HIV involves reversing latency in subjects on suppressive ART [59] and VEH | SIV ECs potently reversed latent HIV to increase the expression of HIV gag p24 in total CD4+ cells (Fig. 7B,C) and in CD25 + CD69+ cells (Fig. 7F,G) within PBMCs of HIV + ART+ donors. Above all, the frequencies of HIV gag p24+ cells within CD25 + CD69 + CD4+ were significantly higher in PBMCs treated with VEH | SIV ECs > VEH ECs > THC | SIV ECs > PBS in that order (Fig. 7H, I).

Unlike VEH | SIV ECs, VEH ECs and THC | SIV ECs did not reactivate latent HIV to express HIV gag p24, but there was a trend towards THC | SIV ECs-mediated increases in CD69 (Fig. 7D, E) and CD25/CD69 (Fig. 7D) expressions, with a trend toward THC | SIV ECs-mediated decreases in CD25 expression (Fig. 7D, E), as previously described [60]. This observation adds to the evidence that the PBMCs were functionally active since the functionality and phenotype of PBMCs post expansion is defined by the expression of CD69 (an early activation antigen involved in transmission of costimulatory signals) and CD25 (part of the IL-2 receptor expressed within 24–48 h after mitogenic stimulation) [61]. Upregulation of CD69/CD25 in PBMCs by stimulation with ECs is significant because CD25 is a reliable indicator of immune activation [62, 63]. Noteworthy, THC induces the expression of CD69 as evidenced by CB1 − / − CB2 −/− mice [60] and as shown in Fig. 7D, and E.

Although ECs generally did not alter cell viability significantly, increased levels of LDH secreted by PBMCs treated with VEH | SIV ECs indicates potential damage and toxicity and in agreement with published studies on LDH as a potential marker for poor prognosis in HIV and comorbidity in PWH [64]. In PWH and non-Hodgkin lymphoma, high LDH levels predicted an unfavorable prognosis after chemotherapy [65] and high LDH levels served as a predictor of mortality in PWH with disseminated histoplasmosis [66]. In acute HIV infection, elevated LDH levels are accompanied by increased viral load [67–69]. In our studies, PBMCs treated with VEH | SIV ECs released significantly higher LDH (Fig. 7A), expressed high HIV gag p24 (Fig. 7C, G), secrete higher HIV gag p24 (Fig. 7J, Supplemental Fig. 9), and produced viral particles that transferred infection to indicator cells (Fig. 7K, L). While the mechanism governing VEH | SIV ECs-induced increase in LDH is unknown, it may be attributed to direct HIV activities, which causes damage and release of LDH into the supernatant [70]. It is also possible that VEH | SIV ECs-induced increase in HIV proteins may lead to interaction of viral proteins with various cellular glycolytic enzymes to increase the glycolytic rate of the PBMCs [71].

While we observed similar effects of ECs on latent HIV reactivation in microglia and PBMCs, the observation in PBMCs is perfectly applicable to HIV reservoir studies focused on the periphery (blood, intestine, lymph nodes, etc.). However, the focus of this study is the brain. Indeed, multiple studies have shown that microglia [72, 73] and not CD4 T cells are the main cell types that harbor the HIV reservoir in the brain. As a result, we believe that the data presented in this study comprehensively address the role of ECs in latent HIV reactivation in the brain (microglial cells). Our approach to focus only on human brain-derived primary microglia is significantly strengthened by the findings of Tang et al. 2023 [73], where the authors, used in vitro viral outgrowth assays, to show that intact HIV particles could be successfully recovered from microglia but not T cells isolated from postmortem brain tissues of people with HIV on long-term antiretroviral therapy. In contrast, in the same study, intact HIV particles were successfully recovered from CD4 T cells isolated from PBMCs. In addition, a recent article from the lab of Dr. Mathias Lichterfeld [72], a leader in the field of HIV latency and persistence, also alluded to the same conclusion that even though CD4 T cells may play a role in early brain infection (Trojan horse), microglia serve as the primary HIV reservoirs in the brain with some contribution from macrophages [72]. Overall, while there is consensus that infected CD4 T cells could transport HIV to the brain and infect myeloid cells during acute infection and some investigators also pointing to the possibility for these cells to clonally expand and persist, no concrete evidence for their role as a long-term HIV brain reservoir, unlike microglia (Tang et al., 2023) [73] is available to this date. This said, we are intrigued to find out if basal ganglia-derived ECs could reactivate latent HIV in primary human PBMC-derived CD4 T cells.

The universality of ECs on latent viruses was assessed using latent EBV infected Akata and Raji B cell lines. VEH ECs and THC | SIV ECs did not induce EBV lytic program that initiates the expression of the viral immediate-early gene BZLF1 (Z) [32–34] and viral promoter BMRF1 [35, 36], which is an early lytic viral protein that functions as the viral DNA polymerase processivity factor [37]. However, VEH | SIV ECs induced EBV lytic program (Supplemental Fig. 8). These findings on ECs-induced EBV reactivation is significant because EBV-infected B and plasma cells can accumulate in the CNS as shown with patients with MS [74, 75], suggesting that ECs can regulate EBV latency but the effect of ECs might depend on their source.

The recognition that ECs (Figs. 1–7**)** isolated from THC treated SIV-infected RMs could suppress virus-induced inflammation or LDH release and inhibit virus production from latently HIV-infected cells emphasizes the importance of understanding the antiviral roles of ECs in the setting of HIV cure research. To the best of our knowledge, the findings of our study represent an initial and the most comprehensive investigation focusing on the potential role of ECs on HIV persistence and the impact of THC in regulating viral persistence via ECs. Future studies are required to identify the cargo of ECs that mediates these effects to better understand the cellular factors that reactivate latently HIV/SIV infected cells in the brain. Additionally, future studies will address the limitations of this study, such as using primary latently HIV-infected cells to assess the effect of ECs on regulating HIV latency and evaluating the effect of human brain derived ECs on HIV latency regulation.

## Methods

Detailed methods are presented in supplemental materials.

### Ethical approvals

Specimens collected with the approval of The University of Iowa Institutional Review Board (IRB) were used. HIV-1 positive subjects consented to participate in this study via written informed consent. All specimens were received unlinked to any identifiers. All experiments were performed in accordance with the approved University guidelines and regulations.

### Purification and characterization of BG ECs

Isolation of ECs was conducted using our previously described protocols where the ECs were formerly called membraneless condensates (MCs) but now called ECs [11, 76]. The basal ganglia derived ECs used in this study were previously characterized by Kaddour et al., 2022 [11]. Detailed method is presented in Supplemental materials and methods.

### Cells lines

TZM-GFP [77]; HIV-1 infected U937 Cells (U1) [78]; J-Lat GFP and J-Lat Tat-GFP cells [79, 80] were obtained through the NIH HIV Reagent Program. CEMx174 cells were provided by co-corresponding author Dr. Mahesh Mohan. Huglia (HC69) cells were a kind gift from Dr. Jonathan Karn. The latent EBV infected Raji and Akata cell lines [81, 82] were kind gifts from Dr. Christopher Whitehurst.

### Internalization of ECs

PBS or ECs were stained with 5 µM SYTO™ RNASelect™ or DiR’; DiIC18(7) (1,1’-Dioctadecyl-3,3,3’,3’-Tetramethylindotricarbocyanine Iodide) [76, 83–85]. Images of the cells were taken at different times, processed (Gen5), and plotted (GraphPad Prism 10.1) [16, 24, 25].

### RT-qPCR

Five µg total RNA was used for cDNA synthesis and real-time PCR (Table 2) using previously described protocol [86].

### Western blot

A total of 50 µg of protein extracts from U1 cells treated with PBS or ECs for 4 days were subjected to 4–20% SDS-PAGE and western blot with relevant antibodies [23, 25, 87]. Blots were processed and images were captured using LI-COR, and band intensity measured using ImageJ [23, 25, 87].

### Viability assay

10,000 cells/well were seeded in 96-well plates and treated with ECs (50 µg/mL) or equivalent volume of 0.1× PBS for 04 days at 37 °C. On day 04 of treatment, cells were counted, and the number of live cells determined using the Luna-II automated cell counter and validated using the trypan blue exclusion assay.

### Cytotoxicity assessment using lactate dehydrogenase (LDH) release assay

The LDH assay was carried out using a commercially available kit according to the manufacturer’s protocol as we previously described [88]. Briefly, at the end of treatment of donor PBMCs with ECs, 50 μL of supernatants from the PBMCs were placed in a 96-well plate in triplicates. Equal volume of LDH reaction mix was added to each well and covered with aluminum foil to protect from light. The plate was incubated at 37 °C for 30 min. After incubation, endpoint absorbance was detected at 490 nm and 680 nm wavelength using the Synergy H1 Mono RDR plate reader.

### RNA-Seq

100-300 µg total RNA were isolated, and RNA quality was assessed by RNA Tapestation and quantified by Qubit 2.0 RNA HS assay [86]. Prior to first strand synthesis, samples were randomly primed (5´ d(N6) 3´ [N = A, C,G,T]) and fragmented based on manufacturer’s recommendations. The first strand is synthesized with the Protoscript II Reverse Transcriptase with a longer extension period, approximately 40 min at 42 °C. All remaining steps for library construction were used according to the NEBNext® Ultra™ II Directional RNA Library Prep Kit for Illumina®. Final libraries quantity was assessed by Qubit 2.0 and quality was assessed by TapeStation D1000 ScreenTape. Equimolar pooling of libraries was sequenced on Illumina® Novaseq platform with a read length configuration of 150 PE for 40 M PE reads/sample (20 M in each direction).

### Bioinformatics

FastQC was applied to check the quality of raw reads. Trimmomatic was applied to cut adaptors and trim low-quality bases with default setting. STAR Aligner was used to align the reads. The package of Picard tools was applied to mark duplicates of mapping. StringTie was used to assemble the RNA-Seq alignments into potential transcripts. FeatureCounts or HTSeq was used to count mapped reads for genomic features such as genes, exons, promoter, gene bodies, genomic bins, and chromosomal locations. Raw TPMs [89, 90] are provided in Supplemental Table 1.

### Secretome analysis

100,000 U1 cells were seeded in a 12 well plate with 500 µL of RPMI media. Cells were treated with ECs (50 µg) or 0.1X PBS for 04 days. On day four, cells were collected, processed, pellets saved for transcriptome analysis. 1 mL of clarified supernatants from each group was used to perform cytokine array analysis with Proteome Profiler Human XL Cytokine Array Kit per manufacturer’s protocol. Target protein expression as Dot blot were quantified using Empiria Studio and protein expression presented as pixel intensity.

### ELISA for extracellular HIV gag p24

To assess the amount of extracellular p24 in the supernatant of PBMCs, we used quantitative ELISA (HIV-1 P24 ELISA, Xpress Bio Cat#XB-1000), following manufacturer’s recommended protocol.

### Syncytia assay

5000 CEMx174 cells/well were seeded in 48 well plate with 250 µL of RPMI media. After two hours, 250 µL of clarified supernatants from PBS or ECs treated U1 cells were added to the cells. Cells were cultured at 37 °C and syncytia were visualized on days 1, 2, and 4 at 10X using Lionheart FX microscope.

### Cell to cell HIV transfer

Cell-to-cell infection of cells was performed with 1000 PKHRed-labeled CEMx174 cells cultured in the presence of 250 µL of clarified supernatants from PBS or ECs treated U1 cells. After four days, the PKHRed-labeled CEMx174 were overlayed atop 1000 TZM-GFP cells and the co-culture incubated for 2 days. The activation of GFP expression in TZM-GFP cells is a sign of virus transfer from CEMx174.

### Validation of transcriptome and secretome data

HC69 Huglia cells were used for data validation with RT-qPCR [86] using total RNA from cells and ELISA [29] using clarified culture supernatants.

### Flow cytometry

Cells of interest were processed, resuspended in MACSQuant running buffer, data was acquired using BD Flow cytometer and analyzed using FlowJo™ as previously described [29].

### Statistical analysis

Significance cutoff was set to fold change (FC) > 1.5 or <-1.5, p-value < 0.05, and FDR < 0.05 [11, 91, 92]. Statistical differences were assessed by one- or two- way ANOVA with Šídák’s or Tukey’s multiple comparisons test, or Binary Student’s t tests (Welch’s correction) using GraphPad Prism 10.6.1. Details of specific statistics are on figure legends, as well as in Tables 2 to 4.

## Supplementary information

Supplemental material

Supplemental Table 1

Supplemental Table 2

Supplemental Table 3

## Data Availability

RNA-Seq dataset can be found at https://www.ncbi.nlm.nih.gov/bioproject/1256456 with BioProject ID: PRJNA1256456. Secretome dataset is included within the article and its additional files.
