# Supplementary material for: Extracellular condensates (ECs) are endogenous modulators of HIV transcription and latency reactivation
Source: Mol Psychiatry. Author manuscript; Available in PMC 2026 May 26. (PMC12999493; doi:10.1038/s41380-025-03354-w)
Supplement: Supplemental materials [file NIHMS2143136-supplement-Supplemental_materials.pdf]

Title: Extracellular condensates (ECs) are endogenous modulators of HIV transcription and latency reactivation

Wasifa Naushad[1], Lakmini S Premadasa[2], Vyshnavi Tallapaneni[1], Bryson C. Okeoma[1], Ashok Chaudhary[3,4], Jack T. Stapleton[3,4], Mahesh Mohan[2]*, Chioma M. Okeoma[1,5]*,

[1]Department of Pathology, Microbiology & Immunology, New York Medical College, Valhalla, NY, USA

[2]Host Pathogen Interaction Program, Southwest National Primate Research Center, Texas Biomedical Research Institute, San Antonio, TX 78227-5302 USA

[3]Department of Internal Medicine, Carver College of Medicine, University of Iowa, 200 Hawkins Drive, Iowa City, IA 52242-1109, USA.

[4]Medical Service, Iowa City Veterans Affairs Medical Center, University of Iowa, 604 Highway 6, Iowa City, IA 52246-2208, US.

[5]Lovelace Biomedical Institute, Albuquerque, NM 87108-5127, USA.

*Corresponding authors

Running title: Extracellular condensates regulate HIV persistence

Supplementary Figures
Figures S1 – S9

A

Cell line	Replication competent virus	Site of mutation	Proviral copies/cell	Reference
J-Lat GFP	No	LTR-GFP	1	Jordan et al., 2001; Jordan et al., 2003.
J-Lat Tat-GFP	No	LTR-Tat-GFP	1	Jordan et al., 2001; Jordan et al., 2003.
U1	Yes	Tat	2	Folks et al., 1989
Huglia (HC69)	Yes	Tat	1	Alvarez-Carbonell et al., 2017

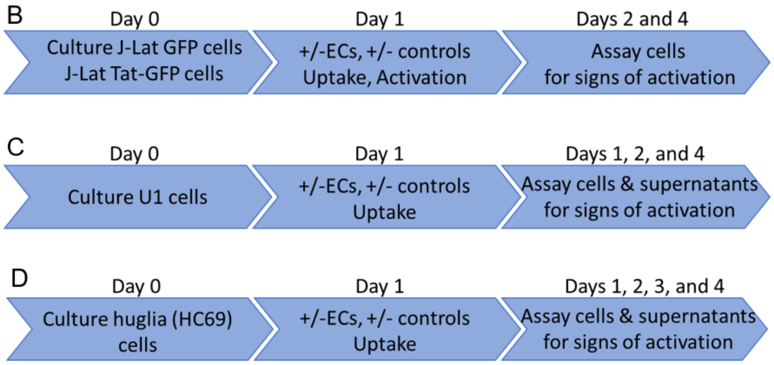

Supplemental Figure 1: A) Description of cell lines for model systems of post-integration latency. **B-D**) Pictorial description of cell types, treatments, and length of treatment and culturing.

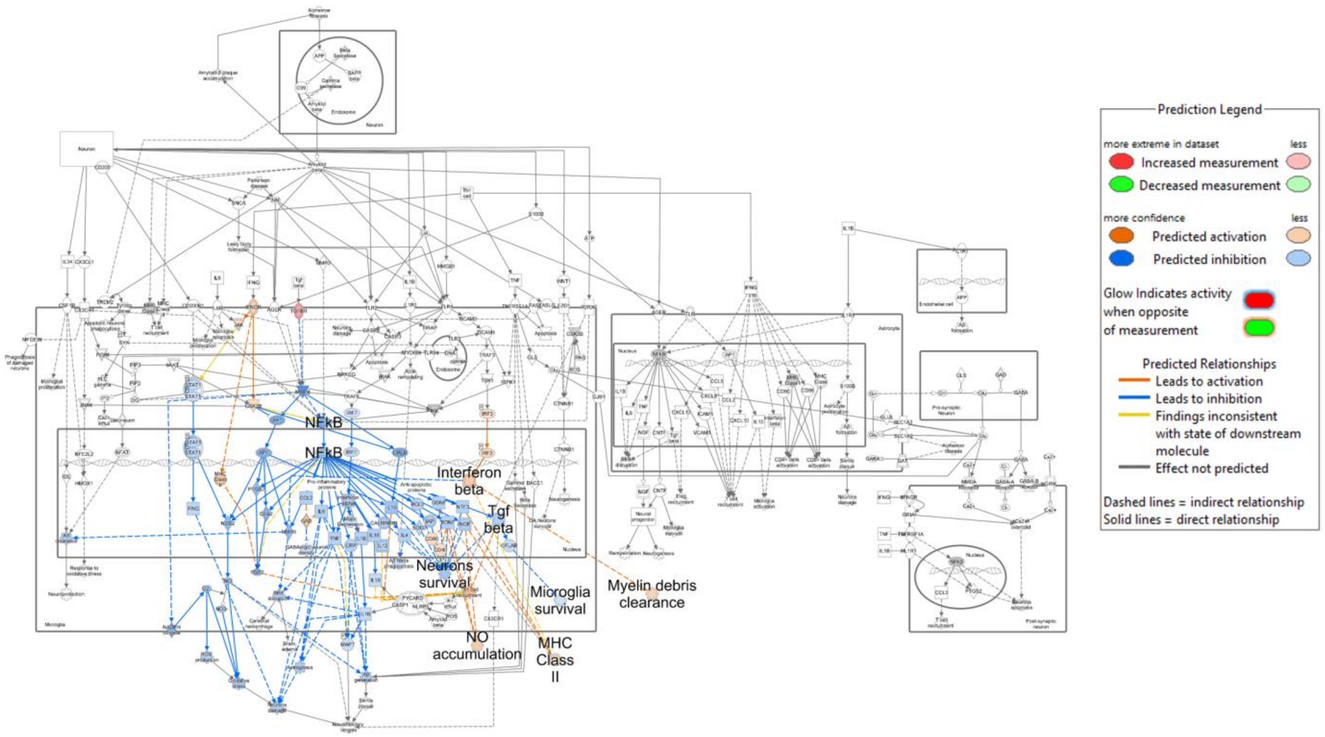

Supplemental Figure 2: Ingenuity Pathway Analysis (IPA) neuroinflammation signaling pathway induced by ECs isolated from control VEH rhesus macaques. Shown are the principal interaction networks in cells treated with VEH ECs.

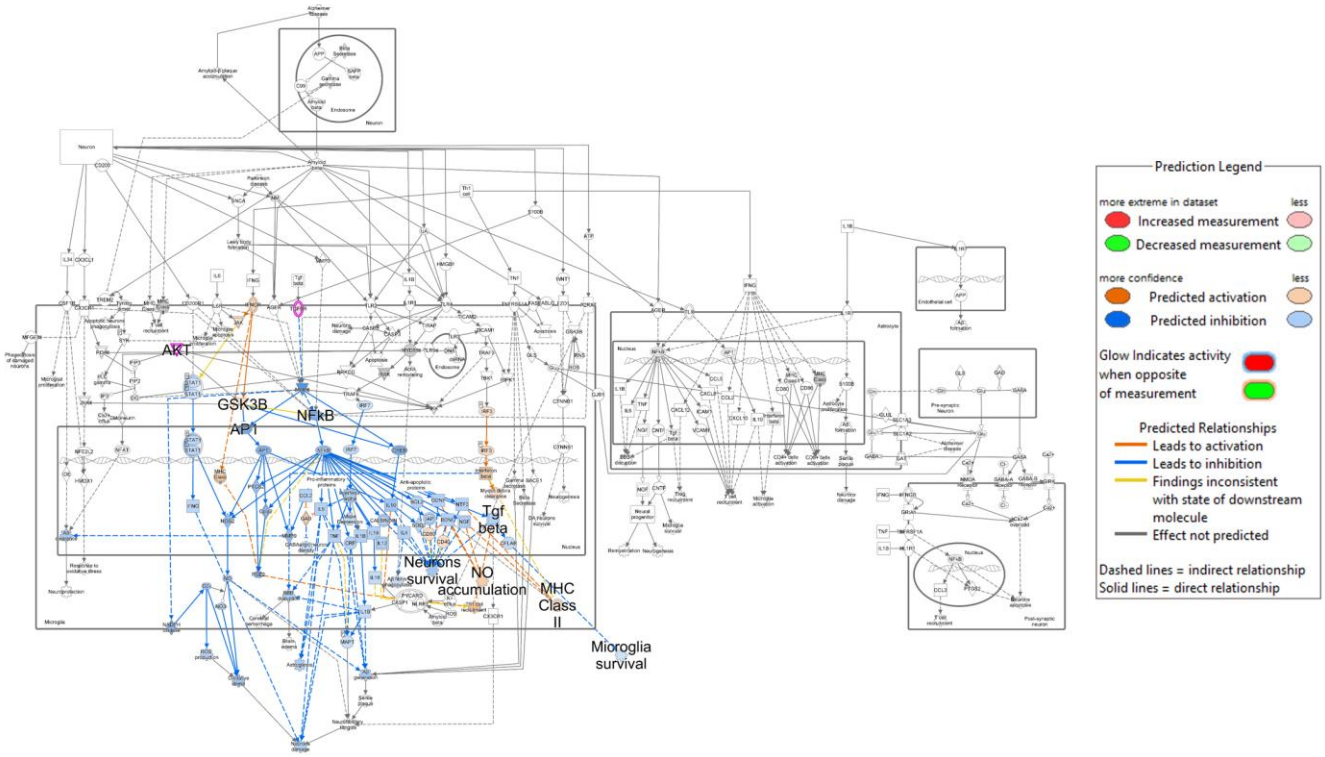

Supplemental Figure 3: Ingenuity Pathway Analysis (IPA) neuroinflammation signaling pathway induced by ECs isolated from SIV-infected (VEH|SIV) rhesus macaques. Shown are the principal interaction networks in cells treated with VEH|SIV ECs.

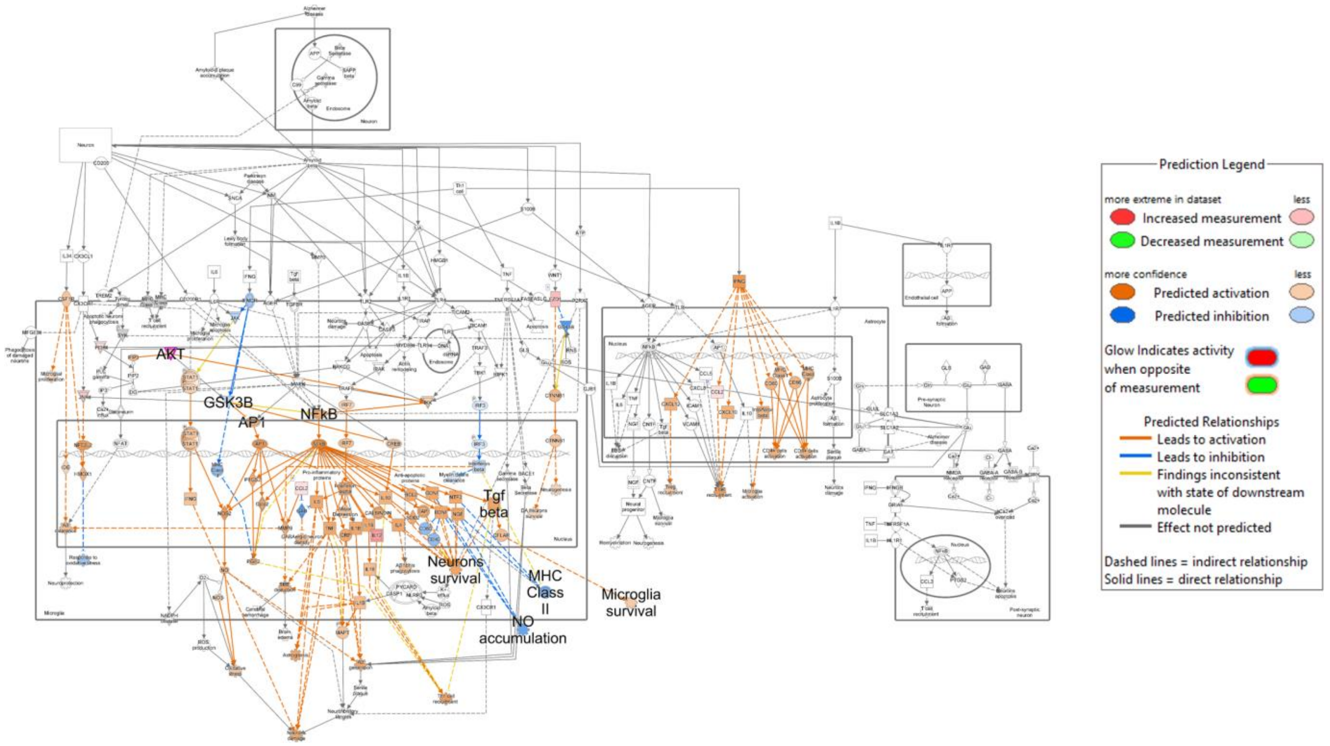

Supplemental Figure 4: Ingenuity Pathway Analysis (IPA) neuroinflammation signaling pathway induced by ECs isolated from THC-treated SIV-infected (THC|SIV) rhesus macaques. Shown are the principal interaction networks in cells treated with THC|SIV ECs.

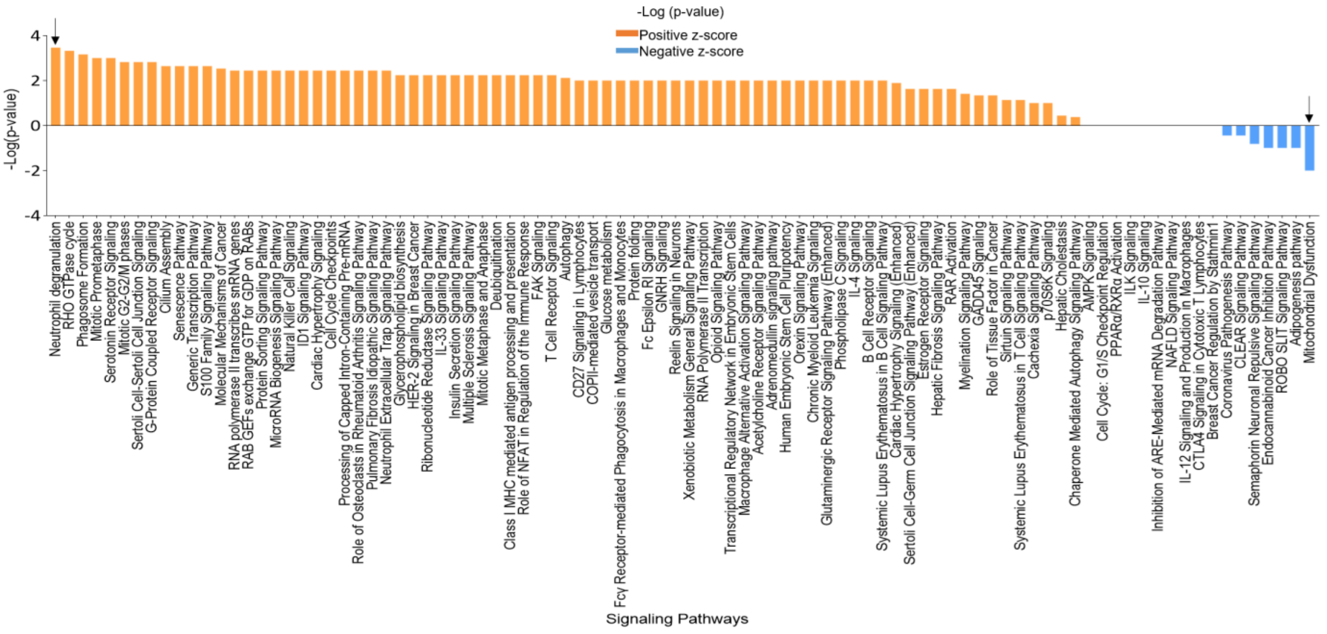

Supplemental Figure 5: Global transcriptome analysis of HIV latently infected myeloid cells treated with basal ganglia derived ECs: Histogram of the 92 canonical pathways regulated by treatment with THC|SIV ECs. The X-axis represents the canonical pathways, and the Y-axis represents -log (P-value) of the canonical pathways. Black arrows point to the most upregulated (neutrophil degranulation) and downregulated (mitochondrial dysfunction) pathways. Each bar indicates the value of -log10 (P-value).

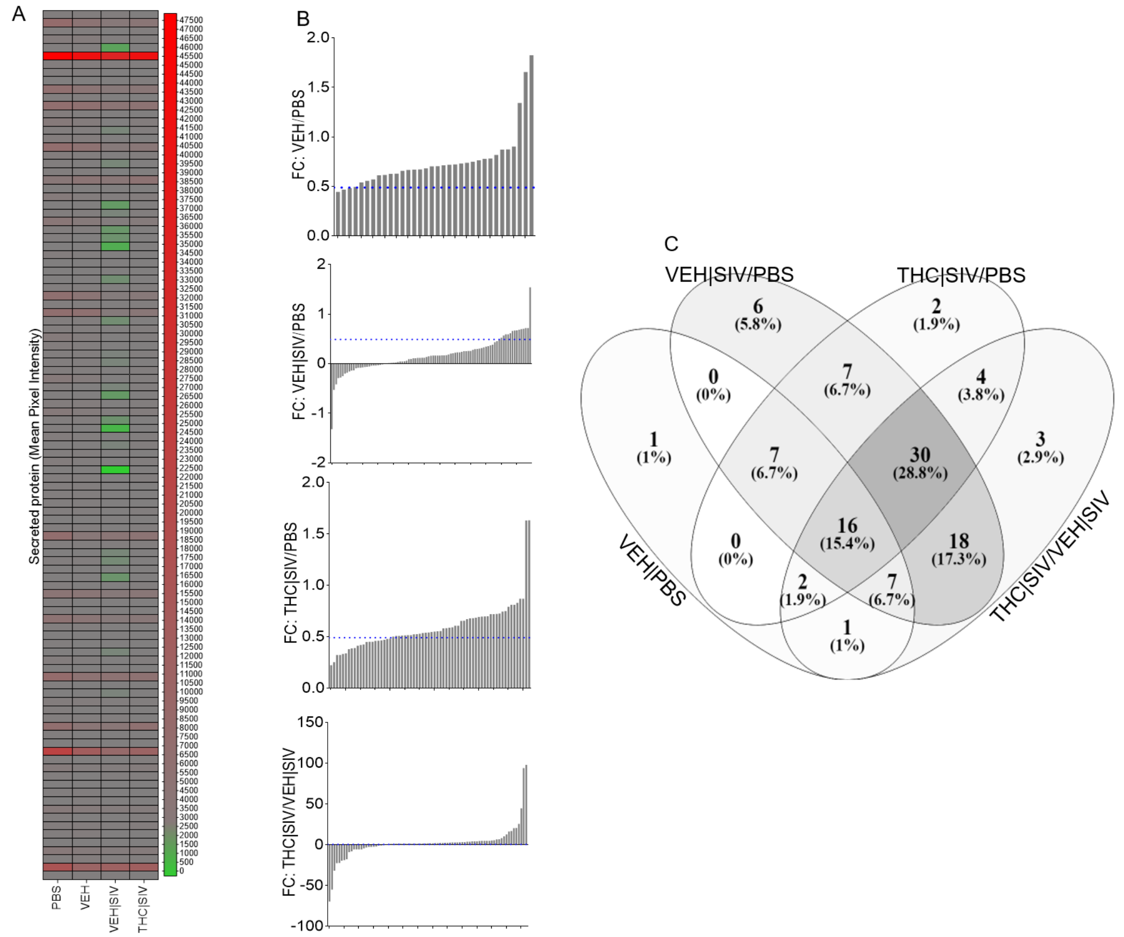

Supplemental Figure 6: Altered secretion of proteins by HIV latently infected U1 cells treated with basal ganglia derived ECs: A) Heatmap of Mean Pixel Intensity of identified secreted proteins. B) Fold change (FC) of secreted proteins between groups. From top to bottom are plots for VEH/PBS (34 proteins), VEH|SIV/PBS (91 proteins), THC|SIV/PBS (68 proteins), THC|SIV/VEH|SIV (81 proteins). The y-axis represents DEG fold change (FC), while the x-axis represents altered DEGs. C) 4-way Venn diagram-based overlap analysis of all secreted proteins showing the number of overlapping and unique proteins.

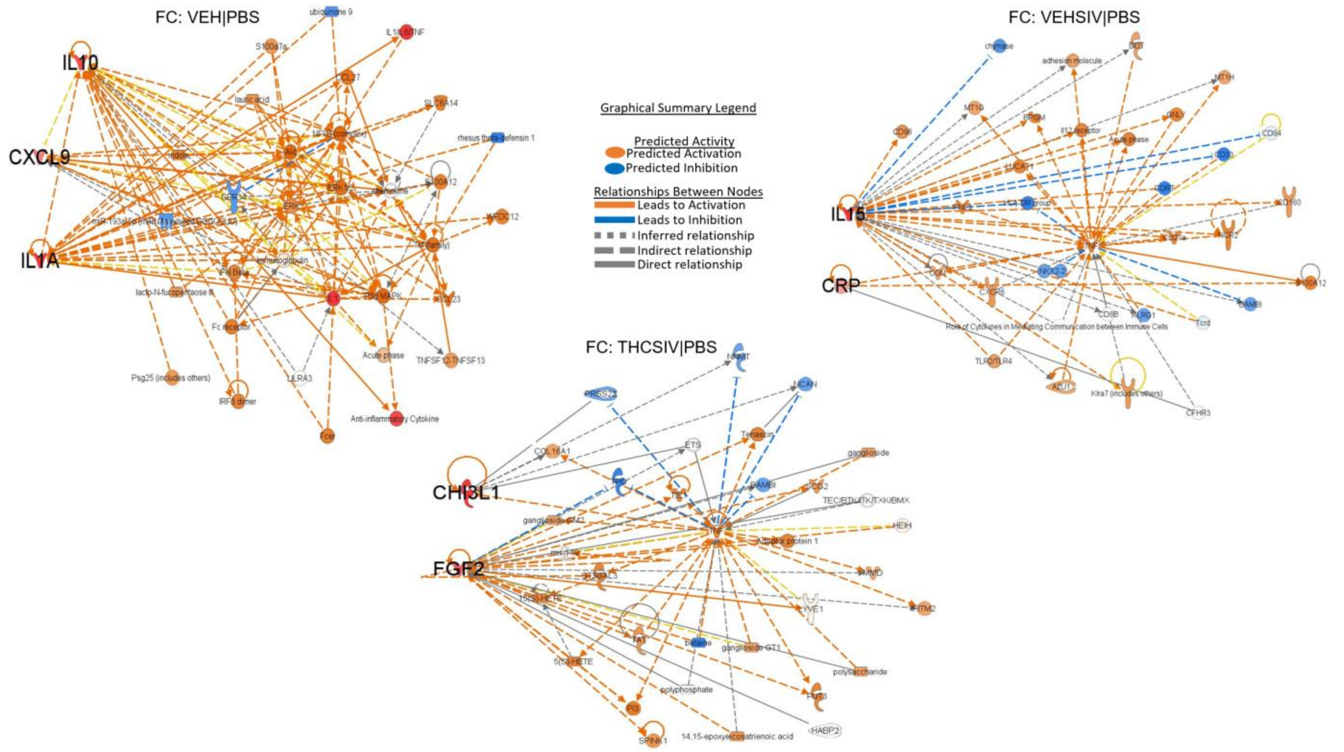

Supplemental Figure 7: Altered secretion of proteins by HIV latently infected U1 cells treated with basal ganglia derived ECs showing IPA network diagrams representing the regulatory networks with top consistency scores found associations with different molecules.

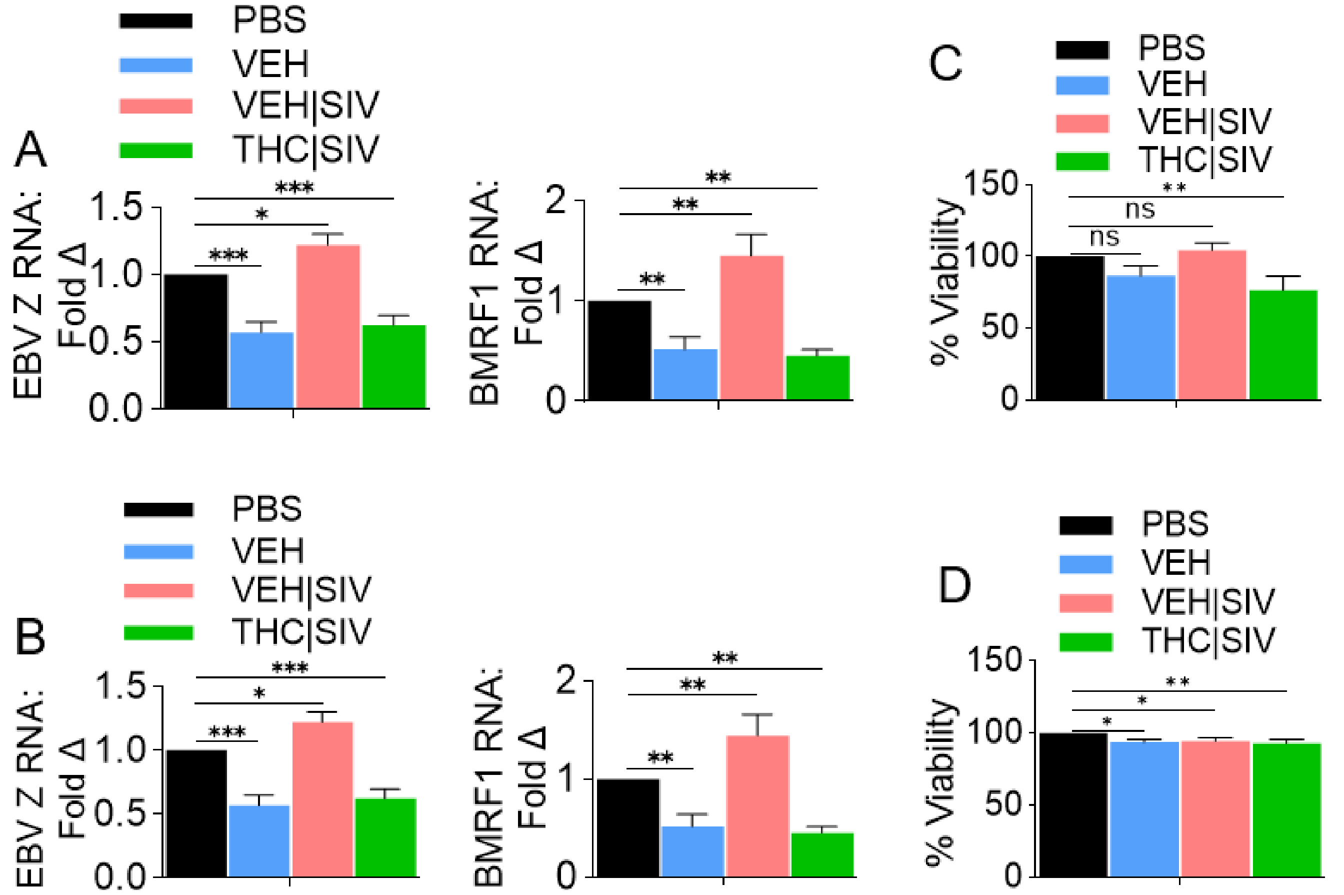

Supplemental Figure 8: Induction of Epstein-Barr virus (EBV) lytic gene expression by VEH|SIV but not THC|SIV ECs: A, B) Realtime PCR analysis of BZLF1 (EBV Z) and BMRF1 expression in Akata (A) and Raji (B) cells. C, D) Viability (MTT) of Akata (C) and Raji (D) cells treated with treated with ECs. Statistical significance was determined by ordinary one-way ANOVA (Šídák's multiple comparisons test) *** p < 0.0005, ** p < 0.0051, * p < 0.01, ns = non-significant.

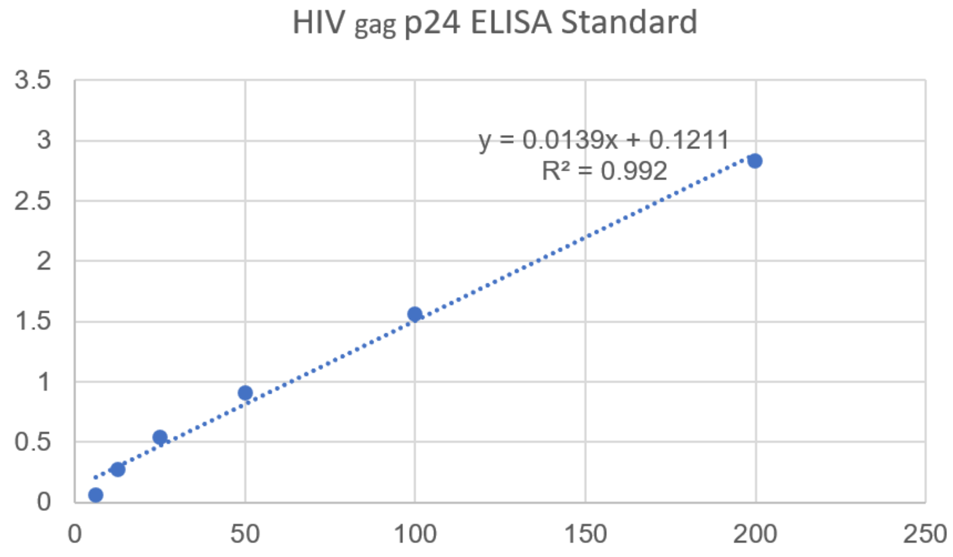

Raw values for HIV gag p24												
	Donor 1 (D1)			Donor 2 (D2)			Donor 3 (D3)			Donor 4 (D4)		
PBS	35.4	54.9	57.2	45.2	40.9	45.2	29.1	37.0	19.1	23.3	20.2	21.2
PMA	91.2	86.4	87.5	48.1	48.9	48.8	37.5	37.5	30.4	35.5	35.3	26.8
VEH	48.9	47.6	45.4	33.5	28.9	40.7	20.3	42.6	25.2	12.6	18.8	10.1
VEH|SIV	87.6	85.8	84.6	56.3	55.7	56.1	34.7	36.9	34.2	41.0	40.5	31.6
VEH|SIV	21.2	22.1	22.4	20.4	20.7	19.0	10.1	9.9	7.3	10.9	12.1	6.3

Supplemental Figure 9: Production of extracellular HIV gag p24 by ELISA: HIV replication was measured by quantifying the accumulation of HIV gag p24 in PBMC culture supernatants by ELISA (HIV-1 p24 ELISA, Xpress Bio Cat#XB-1000)). Top) Standard and Bottom) Raw p24 values extrapolated from the standard curve.

Supplemental Table 1: List of genes with Raw TPMs P values ≥1.

Supplemental Table 2: List of Differentially expressed genes (DEGs) identified using p-value (unpaired t-test) <0.05, p-adj (FDR <0.05).

Supplemental Table 3: List of genes identified with Venn overlap analysis.
