# Supplementary material for: Extracellular condensates (ECs) are endogenous modulators of HIV transcription and latency reactivation
Source: Mol Psychiatry. 2025 Nov 29;31(4):2232–49. doi: 10.1038/s41380-025-03354-w (PMC12999493; doi:10.1038/s41380-025-03354-w)
Supplement: Supplementary file 1 [file 41380_2025_3354_MOESM1_ESM.docx]

**Title: Extracellular condensates (ECs) are endogenous modulators of HIV transcription and latency reactivation**

Wasifa Naushad^1^, Lakmini S Premadasa^2^**,** Vyshnavi Tallapaneni^1^, Bryson C. Okeoma^1^, Ashok Chaudhary^3,4^,, Jack T. Stapleton^3,4^, Mahesh Mohan^2^*, Chioma M. Okeoma^1,5^*,

^1^Department of Pathology, Microbiology & Immunology, New York Medical College, Valhalla, NY, USA

^2^Host Pathogen Interaction Program, Southwest National Primate Research Center, Texas Biomedical Research Institute, San Antonio, TX 78227-5302 USA 

^3^Department of Internal Medicine, Carver College of Medicine, University of Iowa, 200 Hawkins Drive, Iowa City, IA 52242-1109, USA.

^4^​Medical Service, Iowa City Veterans Affairs Medical Center, University of Iowa, 604 Highway 6, Iowa City, IA 52246-2208, US.

^5^Lovelace Biomedical Institute, Albuquerque, NM 87108-5127, USA.

*Corresponding authors

**Running title: Extracellular condensates regulate HIV persistence**

**Methods:**

**Ethical approvals:** Specimens collected with the approval of The University of Iowa Institutional Review Board (IRB) were used.  HIV-1 positive subjects consented to participate in this study via written informed consent. All specimens were received unlinked to any identifiers. All experiments were performed in accordance with the approved University guidelines and regulations.

**Chronic administration of THC or VEH:** Administration of THC and Vehicle (VEH) was initiated by the intramuscular route four weeks before SIV infection at 0.18 mg/kg as used in previous studies [1, 2]. This dose of THC was found to eliminate responding in a complex operant behavioral task in almost all animals [3]. The dose was subsequently increased for each subject to 0.32 mg/kg, over a period of approximately two weeks when responding was no longer affected by 0.18 mg/kg daily (i.e., tolerance developed) and maintained for the duration of the study. The optimization of the THC administration in RMs accounts for the development of tolerance during the initial period of administration. Because in our previously published studies [1, 2] this dose of THC showed protection, the same dose was used in this study. The 0.32 mg/kg dose was also shown to be effective in SIV-infected RMs of Chinese origin [4]. SIV levels in plasma and BG were quantified using the TaqMan One-Step Real-time RT-qPCR assay that targeted the LTR gene [2, 5]. At necropsy, entire BG tissue was collected in RNAlater (Thermo Fisher Scientific). 

**Purification and characterization of basal ganglia derived ECs:** Isolation of ECs was conducted using our previously described protocols where the ECs were formerly called membraneless condensates (MCs) but now called ECs [6, 7]. The basal ganglia derived ECs used in this study were previously characterized by Kaddour et al., 2022 [6]. Briefly, small chunks of RNALater-stored BG tissues, ranging from 35-118 mg, were finely chopped and digested with collagenase III. Samples were clarified and supernatants were purified on a 20 x 0.5 cm Sephadex G-50 size exclusion column, using a particle purification liquid chromatography (PPLC) system as previously described [7]. Fifty fractions of 200 µL were collected, and the 3D UV-Vis (230 – 650 nm) fractionation profiles were recorded. A no-tissue collagenase control was used as background. After background subtraction and PPLC analysis for particle size and concentration, EV-containing fractions were used in prior study [6]. EC-containing fractions were pooled and stored in small aliquots at -80°C and used for this study. 

**Cells lines:** The following reagents were obtained through the HIV Reagent Program (https://www.beiresources.org/HIV.aspx), NIAID, NIH: TZM-GFP Human Cell Line (JC.53 Derived), HRP-20041, contributed by David G. Russell and David W. Gludish [8]; HIV-1 Infected U937 Cells (U1), ARP-165 contributed by Folks et al., 1989 [9]; J-Lat GFP (ARP-9856) and J-Lat Tat-GFP (ARP-9851) cells were contributed by Dr. Eric Verdin [10, 11]. Huglia (HC69) and CEMx174 cells were kind gifts from Dr. Jonathan Karn and Dr. Mahesh Mohan respectively. Huglia (HC69) cells were maintained in Dulbecco's modified Eagle's medium (DMEM) (Gibco-BRL/Life Technologies) with low glucose, 1% fetal bovine serum (FBS), 1X glutamine, 50 ug/ml normocin and 1 µM of Dexamethasone. TZM-GFP cells were also maintained in DMEM but with 10% FBS.  U1, J-Lat and CEMx174 cells were grown in RPMI (Gibco-BRL/Life Technologies) containing 5% exosome-depleted FBS (Gibco), 100 U/ml penicillin, 100 μg/ml streptomycin, sodium pyruvate, and 0.3 mg/ml l-glutamine (Invitrogen, Molecular Probes). Raji, a Burkitt's lymphoma cell line latently infected with EBV [12, 13] and Akata - another EBV-positive cell line derived from Burkitt's lymphoma and can be induced to make virus [14] were both cultured in RPMI 1640 media supplemented with 10% FB. Prior to induction of viral gene expression, 100,000 cells were seeded in each well. 24 hours later, cells were treated with PBS, VEH ECs, and VEH|SIV ECs, THC|SIV ECs or with PMA (50 ng/mL) + Sodium butyrate (3 µM/mL) and cultured for 48 hours.

**EC internalization/Uptake:** PBS (control) or ECs were stained with SYTO™ RNASelect™ Green Fluorescent or DiR (DiIC18(7); 1,1′-dioctadecyl-3,3,3′,3′-tetramethylindotricarbocyanine iodide) red fluorescent using a final concentration of 5 µM of dye. PBS (control) or ECs were incubated for 20 minutes at 37°C and purified using Exosome Spin Columns. Labelled ECs were added to cells for imaging using Lionheart FX automated scope. Images were acquired at 10X magnification at 0, 6 and 24 hours. Images were processed using Gen5 and raw data were plotted using GraphPad Prism 10.1 (La Jolla, CA, USA, www.graphpad.com).

**Real-time quantitative PCR (RT-qPCR):** 100,000 cells cultured overnight were treated with PBS or with 100 µg/mL of ECs for 04 days. Five µg total RNA was used for cDNA synthesis using the High-Capacity cDNA Reverse Transcription Kit (Applied Biosystems, Thermofisher). The cDNA was stored at −20 °C until use. The thermal cycler program and expression calculation was setup in a 7500 FAST real-time PCR system (Applied Biosystems, Thermofisher), and the fold change in gene expression was calculated using the standard ∆∆CT method as previously described [15-20].

**Western Blot Analysis of Viral Gene Expression:** A total of 50 µg of protein extracts from U1 cells treated with PBS and ECs for 4 days were subjected to 4–20% SDS-PAGE. The proteins were then transferred to a PVDF membrane, the membrane blocked with 5% BSA in 1× TBST buffer and incubated with relevant primary antibodies at 4 °C overnight. The blot was rinsed with 1× TBST 3–5 times for 5 min and incubated with relevant secondary antibodies for 1 h at room temperature. The blot was rinsed with 1× TBST 3–5 times for 5 min and images were captured using the Odyssey infrared imaging system (LI-COR). The integrated density of the band was measured using ImageJ 1.52a software.

**Viability Assays – Trypan blue exclusion and LDH:** Cells (10,000 cells/well) were seeded in a 96-well plate. Cells were treated with ECs (50 µg/mL) or an equivalent volume of 0.1× PBS for 04 days at 37 °C. On day 04 of treatment, cells were counted, and the number of live cells determined using the Luna-II automated cell counter. In parallel, cell viability was determined using the trypan blue exclusion assay. LDH assay used to evaluate the viability of PBMCs was carried out using a commercially available kit according to the manufacturer’s protocol as we previously described [21]. Briefly, at the end of treatment of donor PBMCs with ECs, 50 μL of supernatants from the PBMCs were placed in a 96-well plate in triplicates. Equal volume of LDH reaction mix was added to each well and covered with aluminum foil to protect from light. The plate was incubated at 37 °C for 30 minutes. After incubation, endpoint absorbance was detected at 490 nm and 680 nm wavelength using the Synergy H1 Mono RDR plate reader.

**RNA-Seq:** Isolated RNA (100-300 µg) sample quality was assessed by RNA Tapestation (Agilent Technologies Inc., California, USA) and quantified by Qubit 2.0 RNA HS assay (ThermoFisher, Massachusetts, USA). Paramagnetic beads coupled with oligo d(T)25 were combined with total RNA to isolate poly(A)+ transcripts based on NEBNext® Poly(A) mRNA Magnetic Isolation Module manual (New England BioLabs Inc., Massachusetts, USA). Prior to first strand synthesis, samples were randomly primed (5´ d(N6) 3´ [N=A,C,G,T]) and fragmented based on manufacturer’s recommendations. The first strand is synthesized with the Protoscript II Reverse Transcriptase with a longer extension period, approximately 40 minutes at 42⁰C. All remaining steps for library construction were used according to the NEBNext® Ultra™ II Directional RNA Library Prep Kit for Illumina® (New England BioLabs Inc., Massachusetts, USA). Final libraries quantity was assessed by Qubit 2.0 (ThermoFisher, Massachusetts, USA) and quality was assessed by TapeStation D1000 ScreenTape (Agilent Technologies Inc., California, USA). The final library size was about 430bp with an insert size of about 300bp. Illumina® 8-nt dual-indices were used. Equimolar pooling of libraries was performed based on quality control (QC) values and sequenced on an Illumina® Novaseq platform (Illumina, California, USA) with a read length configuration of 150 PE for 40M PE reads per sample (20M in each direction).

**Bioinformatics:** FastQC (version v0.12.1) was applied to check the quality of raw reads. Trimmomatic (version v0.39) was applied to cut adaptors and trim low-quality bases with default setting. STAR Aligner (version 2.7.10b) was used to align the reads. The package of Picard tools (version 3.0.0) was applied to mark duplicates of mapping. StringTie (version 2.2.1) was used to assemble the RNA-Seq alignments into potential transcripts. FeatureCounts (version 2.0.6) or HTSeq (version 2.0.3) was used to count mapped reads for genomic features such as genes, exons, promoter, gene bodies, genomic bins, and chromosomal locations. The TPM normalization method was used to report results because TPM results are sample independent,  respects the average invariance, eliminates statistical biases inherent in other measure [22], and the TPMs are guaranteed to be the same across samples [23, 24]. Raw TPMs are provided in **Supplemental Table 1**. 

**Secretome analysis using Cytokine Array/Immunoassay:**  To determine the effect of ECs on the secretome of HIV latently infected cells, 100,000 U1 cells were counted and seeded in a 12 well plate with 500 ul of RPMI media. Cells were treated with 50 ug of ECs or 0.1X PBS for 04 days. Following the fourth day, cells were collected, triplicates were pooled and centrifuged at 500g for 5 minutes. Pellets were saved for other experiments (Transcriptome analysis) and supernatants were transferred to a fresh tube. Supernatants were clarified by centrifuging at 2000g for 10 minutes. Clarified supernatants were stored at -80 ^o^C for further analysis. 1 mL of supernatants from each group was used to perform cytokine array analysis following the manufacturer’s protocol (Proteome Profiler Human XL Cytokine Array Kit Catalog Number ARY022B). [https://resources.rndsystems.com/pdfs/datasheets/ary022b.pdf?v=20231215](https://resources.rndsystems.com/pdfs/datasheets/ary022b.pdf?v=20231215). Images of the membranes were acquired using Odyssey infrared imaging system Li-Cor CLX. Target protein expression as Dot blot were quantified using Empiria Studio ® Version 3.0 and protein expression presented as pixel intensity.

**Syncytia Formation Assay:** Syncytia assay was conducted using CEMx174 cells. Briefly, equivalent numbers (5000 cells/well) of CEMx174 cells were seeded in 48 well plate with 250 µl of RPMI media for 2 hours. After 2 hours, 250 µl of clarified of supernatants from U1 cells treated with PBS, or 50 µg of ECs for 04 days were added to the cells. The cells were cultured at 37 °C. After 1, 2, and 4 days of culture, syncytia were visualized and imaged (10X) using Lionheart FX microscope. 

**Cell to cell HIV transfer assay:** Cell-to-cell infection of TZM-GFP cells was performed with CEMx174 cells. Briefly, CEMx174 cells were labeled with PKHRed. The cells were with 1X DPBS to remove excess PKHRed label. Washed CEMx174 cells were cultured in the presence of supernatants from U1 cells treated with PBS or 50 ug of ECs for 04 days. Then, 1000 of the PKHRed-labeled CEMx174 cells were overlayed atop 1000 TZM-GFP cells. The co-culture was incubated for 02 days. Images of the cells were taken with LionHeart FX microscope at 10X magnification and analyzed with Gen5 3.10. The activation of GFP expression in TZM-GFP cells is a sign of virus transfer from CEMx174. 

**Validation of transcriptome and secretome data:** Select genes from the transcriptome dataset were validated using total RNA isolated from independent experiments. The RNA samples were subjected to RT-qPCR and described above. On the other hand, validation of secretome data was conducted using ELISA [19]. Briefly, supernatants from HC69 Huglia cells were used to validate secretome cytokine array analysis. Briefly, 100,000 cells/well were plated overnight in a 12 well plate in 500 µl of DMEM-F12 supplemented with 1% FBS, and 1uM of Dexamethasone. The next day, cells were treated with ECs (50 µg/mL) or 0.1X PBS and incubated at 37^o^C for 04 days. Images were acquired each day to measure GFP intensity. At 04 day, supernatants were collected and clarified by centrifuging at 2000g for 10 minutes at 4^o^C and stored at -80^ o^C freezer for future use. To perform ELISA, high binding capacity 96 well plates (Costar 3166) were used. Multiple dilutions (1:1, 1:2, and 1:4) of HC69 supernatants (antigen) to coating buffer (100 mM of carbonate buffer) were made. Plates were coated with 100 µl/well antigen:coating buffer mix and incubated at 4^o^C overnight. For negative control, media:coating buffer (1:1) was used to coat the wells. Next day, plates were washed 3 times with ELISA wash buffer (Tris buffered saline with Tween 20, pH 8) and residual buffer removed by inverting the plate on paper towels. After washing, 100 µl of blocking buffer (5% BSA with NP-40) was added and plates were incubated at room temperature for 03 hours. After 03 hours, the plates were washed (as stated above), primary antibody (0.2 µg/mL) was added, and plates incubated at 4^o^C overnight. The next day, plates were washed and specie-specific HRP conjugated secondary antibody (1:10,000) was added, plates incubated at room temperature for 03 hours, washed, and excess media removed. 100 µl of TMB substrate (Abcam ab171523) was added to each well. After 15 minutes, stop solution (0.2M sulfuric acid) was added to each well. Absorbance was read at 450nm using Biotek synergy H1 reader. 

**Validation of transcriptome and secretome data:** HC69 Huglia cells were used for data validation. Select genes from the transcriptome dataset were validated with RT-qPCR [25] using total RNA isolated from huglia cells, while secretome data were validated with supernatants for the huglia cells using ELISA [19].

**Primary PBMC reactivation:** Cryopreserved PBMCs from 4 donors (**Table 8**) were rested in RPMI 1640 supplemented with 10% FBS, penicillin [50 U/ml], streptomycin [50 μg/ml], and L-glutamine [2 mM]) at a concentration of 1×10^6^/mL. On day 02, the cells were stimulated with 10 μg/mL PHA in the presence of 100 IU/mL IL-2 (Life Technologies). On day 07, the cells were harvested, counted and plated at a concentration of 1×10^6^ cells/mL. For the treatment groups, the cells were treated with PBS and ECs at a concentration of 50 µg/mL. PMA (10 ng/mL) was added to cells as a positive control group [19]. After 96 h of treatment, the cells were harvested. The cells were used for flow cytometry analysis using previously published protocol [19]. Clarified supernatants were used for LDH cytotoxicity and viral outgrowth assays.  

**Flow Cytometry:** For J-Lat -GFP and J-Lat Tat-GFP cells, equivalent numbers (100,000) of J cells were seeded in 48 well plate containing 500 µl of media. The cells were treated with 0.1X PBS or 50 µg/mL VEH, VEH|SIV, THS|SIV ECs. Cells were cultured in the presence of PBS or ECs for 04 days. On day 04, cells were washed and resuspended in MACSQuant running buffer (Miltenyi Biotec) to perform flow cytometry using BD Flow cytometer. Analysis was performed using FlowJo™ v10.10.  For primary PBMCs, cells were fixed with 4% PFA, permeabilized with 100 % methanol, washed with PBS, and stained with antibodies against human CD4 Percp-CY5.5 50TST (BDB560650), CD25 mouse anti-human Brilliant Violet 605 (Clone: 2A3, BD BDB562661), CD69 monoclonal antibody [(FN50) APC, eBioscience™, Invitrogen™ 50-150-17], and anti-HIV p24 monoclonal KC57 FITC ( NIH ARP Cat:13450). Cells were washed and resuspended in MACSQuant running buffer (Miltenyi Biotec) and analysis performed using BD flow cytometer and data analyzed using FloJo [19]. 

**ELISA for extracellular HIV gag p24:** To assess the amount of extracellular p24 in the supernatant of PBMCs, we used quantitative ELISA (HIV-1 P24 ELISA, Xpress Bio Cat#XB-1000), following manufacturer’s recommended protocol. Thus, culture supernatants were collected and concentrated (using Eppendorf Concentrator plus) from a total volume 500 µL to 300 µL. All controls and samples were tested in triplicates. 200 pg/mL p24 was used as positive control while culture media was used as a negative control and reagent blank. 200 µL of each control and specimen was dispensed into appropriate wells of precoated microplate and incubated at 37 °C for 60 minutes. Then the contents of the wells were aspirated and the microtitration plate was washed with wash buffer provided with the kit. Then 100 µL of detection antibody was added into each well and incubate at 37 °C for 60 minutes. The detector antibody was also aspirated from the wells and the microtitration plate was washed with wash buffer. 100 µL of Streptavidin HRP conjugate was added into each well and incubated at room temperature (18 – 25 °C) for 30 minutes. Later, the conjugate was washed from the wells and without delay, 100 µL Substrate Solution was dispensed into each well and incubated at room temperature (18 – 25 °C) protected from direct sunlight, for 30 minutes. To Stop the reaction, 100 µL of Stop Solution was added to each well including the reagent blank and negative control and the absorbance values were read at 450 nm using a H-Synergy microplate reader.

**Statistical analysis:** Differential expression data were generated using Graphpad Prism. The significance cutoff was set to fold change (FC) >1.5 or <-1.5 and a p-value <0.05. Statistical differences were assessed by one-way ANOVA with Šídák's multiple comparisons test, two-way ANOVA with Tukey’s multiple comparison test, or Binary Student’s t tests (Welch’s correction) using GraphPad Prism 10. Details of specific statistics are presented in each figure legend where the p-values are listed. 

**Abbreviations:**

ART – Antiretroviral therapy

BG – Basal ganglia

CNS – Central nervous system

EBV – Epstein-Barr Virus

ECs – Extracellular condensates

EVs – Extracellular vesicles

HIV, HIV-1 – human immunodeficiency virus type 1

NVEPs – Non-vesicular Extracellular Particles 

SIV – simian immunodeficiency virus 

THC – Delta-9-tetrahydrocannabinol

VEH – Vehicle

**References:**

1.	Chandra LC, Kumar V, Torben W, Vande Stouwe C, Winsauer P, Amedee A *et al.* Chronic administration of Delta9-tetrahydrocannabinol induces intestinal anti-inflammatory microRNA expression during acute simian immunodeficiency virus infection of rhesus macaques. *J Virol* 2015; **89**(2)**:** 1168-1181.

2.	Kumar V, Torben W, Mansfield J, Alvarez X, Vande Stouwe C, Li J *et al.* Cannabinoid Attenuation of Intestinal Inflammation in Chronic SIV-Infected Rhesus Macaques Involves T Cell Modulation and Differential Expression of Micro-RNAs and Pro-inflammatory Genes. *Front Immunol* 2019; **10:** 914.

3.	Winsauer PJ, Molina PE, Amedee AM, Filipeanu CM, McGoey RR, Troxclair DA *et al.* Tolerance to chronic delta-9-tetrahydrocannabinol (Delta(9)-THC) in rhesus macaques infected with simian immunodeficiency virus. *Exp Clin Psychopharmacol* 2011; **19**(2)**:** 154-172.

4.	Wei Q, Liu L, Cong Z, Wu X, Wang H, Qin C *et al.* Chronic Delta(9)-Tetrahydrocannabinol Administration Reduces IgE(+)B Cells but Unlikely Enhances Pathogenic SIVmac251 Infection in Male Rhesus Macaques of Chinese Origin. *J Neuroimmune Pharmacol* 2016; **11**(3)**:** 584-591.

5.	McDew-White M, Lee E, Alvarez X, Sestak K, Ling BJ, Byrareddy SN *et al.* Cannabinoid control of gingival immune activation in chronically SIV-infected rhesus macaques involves modulation of the indoleamine-2,3-dioxygenase-1 pathway and salivary microbiome. *EBioMedicine* 2022; **75:** 103769.

6.	Kaddour H, McDew-White M, Madeira MM, Tranquille MA, Tsirka SE, Mohan M, Okeoma CM. Chronic delta-9-tetrahydrocannabinol (THC) treatment counteracts SIV-induced modulation of proinflammatory microRNA cargo in basal ganglia-derived extracellular vesicles. *J Neuroinflammation* 2022; **19**(1)**:** 225.

7.	Kaddour H, Lyu Y, Shouman N, Mohan M, Okeoma CM. Development of Novel High-Resolution Size-Guided Turbidimetry-Enabled Particle Purification Liquid Chromatography (PPLC): Extracellular Vesicles and Membraneless Condensates in Focus. *Int J Mol Sci* 2020; **21**(15).

8.	Gludish DW, Boliar S, Caldwell S, Tembo DL, Chimbayo ET, Jambo KC *et al.* TZM-gfp cells: a tractable fluorescent tool for analysis of rare and early HIV-1 infection. *Sci Rep* 2020; **10**(1)**:** 19900.

9.	Folks TM, Clouse KA, Justement J, Rabson A, Duh E, Kehrl JH, Fauci AS. Tumor necrosis factor alpha induces expression of human immunodeficiency virus in a chronically infected T-cell clone. *Proc Natl Acad Sci U S A* 1989; **86**(7)**:** 2365-2368.

10.	Jordan A, Bisgrove D, Verdin E. HIV reproducibly establishes a latent infection after acute infection of T cells in vitro. *Embo j* 2003; **22**(8)**:** 1868-1877.

11.	Jordan A, Defechereux P, Verdin E. The site of HIV-1 integration in the human genome determines basal transcriptional activity and response to Tat transactivation. *Embo j* 2001; **20**(7)**:** 1726-1738.

12.	Pulvertaft JV. CYTOLOGY OF BURKITT'S TUMOUR (AFRICAN LYMPHOMA). *Lancet* 1964; **1**(7327)**:** 238-240.

13.	Epstein MA, Barr YM. CHARACTERISTICS AND MODE OF GROWTH OF TISSUE CULTURE STRAIN (EB1) OF HUMAN LYMPHOBLASTS FROM BURKITT'S LYMPHOMA. *J Natl Cancer Inst* 1965; **34:** 231-240.

14.	Wang FZ, Roy D, Gershburg E, Whitehurst CB, Dittmer DP, Pagano JS. Maribavir inhibits epstein-barr virus transcription in addition to viral DNA replication. *J Virol* 2009; **83**(23)**:** 12108-12117.

15.	Lyu Y, Kopcho S, Mohan M, Okeoma CM. Long-Term Low-Dose Delta-9-Tetrahydrocannbinol (THC) Administration to Simian Immunodeficiency Virus (SIV) Infected Rhesus Macaques Stimulates the Release of Bioactive Blood Extracellular Vesicles (EVs) that Induce Divergent Structural Adaptations and Signaling Cues. *Cells* 2020; **9**(10).

16.	Madison MN, Jones PH, Okeoma CM. Exosomes in human semen restrict HIV-1 transmission by vaginal cells and block intravaginal replication of LP-BM5 murine AIDS virus complex. *Virology* 2015; **482:** 189-201.

17.	Madison MN, Roller RJ, Okeoma CM. Human semen contains exosomes with potent anti-HIV-1 activity. *Retrovirology* 2014; **11:** 102.

18.	Welch JL, Kaddour H, Schlievert PM, Stapleton JT, Okeoma CM. Semen exosomes promote transcriptional silencing of HIV-1 by disrupting NF-kB/Sp1/Tat circuitry. *J Virol* 2018.

19.	Welch JL, Kaufman TM, Stapleton JT, Okeoma CM. Semen exosomes inhibit HIV infection and HIV-induced proinflammatory cytokine production independent of the activation state of primary lymphocytes. *FEBS Lett* 2020; **594**(4)**:** 695-709.

20.	Welch JL, Madison MN, Margolick JB, Galvin S, Gupta P, Martínez-Maza O *et al.* Effect of prolonged freezing of semen on exosome recovery and biologic activity. *Sci Rep* 2017; **7:** 45034.

21.	Mohammed S, Shamseddine AA, Newcomb B, Chavez RS, Panzner TD, Lee AH *et al.* Sublethal doxorubicin promotes migration and invasion of breast cancer cells: role of Src Family non-receptor tyrosine kinases. *Breast Cancer Res* 2021; **23**(1)**:** 76.

22.	Wagner GP, Kin K, Lynch VJ. Measurement of mRNA abundance using RNA-seq data: RPKM measure is inconsistent among samples. *Theory Biosci* 2012; **131**(4)**:** 281-285.

23.	Dillies MA, Rau A, Aubert J, Hennequet-Antier C, Jeanmougin M, Servant N *et al.* A comprehensive evaluation of normalization methods for Illumina high-throughput RNA sequencing data analysis. *Brief Bioinform* 2013; **14**(6)**:** 671-683.

24.	Fundel K, Haag J, Gebhard PM, Zimmer R, Aigner T. Normalization strategies for mRNA expression data in cartilage research. *Osteoarthritis Cartilage* 2008; **16**(8)**:** 947-955.

25.	Kaddour H, Kopcho S, Lyu Y, Shouman N, Paromov V, Pratap S *et al.* HIV-infection and cocaine use regulate semen extracellular vesicles proteome and miRNAome in a manner that mediates strategic monocyte haptotaxis governed by miR-128 network. *Cell Mol Life Sci* 2021; **79**(1)**:** 5.
